# Tuning the Properties of Dodecylpyridinium Metallosurfactants: The Role of Iron-Based Counterions

**DOI:** 10.3390/ijms26062540

**Published:** 2025-03-12

**Authors:** Mirta Rubčić, Mirta Herak, Ana Ivančić, Edi Topić, Emma Beriša, Ivana Tartaro Bujak, Darija Domazet Jurašin

**Affiliations:** 1 Department of Chemistry, Faculty of Science, University of Zagreb, Horvatovac 102a, HR-10000 Zagreb, Croatia; mirta@chem.pmf.hr (M.R.); edi.topic@chem.pmf.hr (E.T.); 2 Department for Research of Materials Under Extreme Conditions, Institute of Physics, Bijenička Cesta 46, HR-10000 Zagreb, Croatia; mirta@ifs.hr; 3 Division of Physical Chemistry, Ruđer Bošković Institute, Bijenička 54, HR-10000 Zagreb, Croatia; aivancic@irb.hr; 4 Faculty of Chemical Engineering and Technology, University of Zagreb, Trg Marka Marulića 19, HR-10000 Zagreb, Croatia; eberisa@fkit.hr; 5 Division of Materials Chemistry, Ruđer Bošković Institute, Bijenička 54, HR-10000 Zagreb, Croatia; itartaro@irb.hr

**Keywords:** metallosurfactants, inorganic-organic hybrids, iron complexes, crystal structure, solid-state magnetism, low-dimensional magnets, self-assembly, ionic liquids

## Abstract

Metallosurfactants combine the unique soft-matter properties of surfactants with magnetic functionalities of metal ions. The inclusion of iron-based species, in particular, can further boost the functionality of the material, owing to iron’s ability to adopt multiple oxidation states and form both high-spin and low-spin complexes. Motivated by this, a series of hybrid inorganic-organic dodecylpyridinium metallosurfactants with iron-containing counterions was developed. It was established that using either divalent or trivalent iron halides in a straightforward synthetic procedure yields C_12_Py-metallosurfactants with distinct complex counterions: (C_12_Py)_2_[Fe_2_X_6_O] and (C_12_Py)[FeX_4_] (X = Cl or Br), respectively. A combination of techniques—including conductometry, dynamic and electrophoretic light scattering, single-crystal and thermogravimetric analysis, and magnetic measurements—provided in-depth insights into their solution and solid-state properties. The presence of different iron-based counterions significantly influences the crystal structure (interdigitated vs. non-interdigitated bilayers), magnetic properties (paramagnetic vs. nonmagnetic singlet ground state), and self-assembly (vesicles vs. micelles) of the dodecylpyridinium series. To our knowledge, this is the first report on the synthesis and characterization of hybrid organic-inorganic metallosurfactants containing the *μ*-oxo-hexahalo-diferrate anion.

## 1. Introduction

In today’s era of rapid technological advancements, developing cost-effective, environmentally friendly, and multifunctional materials has become the top priority of materials science. Surfactants offer vast potential in this context, due to their unique physicochemical properties in both solution and in the solid state.

Among various surfactant classes, metallosurfactants have gained considerable attention over the past decade [1,2,3]. These compounds combine surfactants’ amphiphilic nature with metal-containing complexes’ distinctive properties by incorporating transition or inner-transition metals as structural components. The presence of metal ions further endows such systems with variable oxidation states, color tunability, magnetism, and pH sensitivity, while retaining intrinsic adsorption and self-assembly abilities [1,2,3]. Within this class, magnetic surfactants exhibiting magneto-responsive properties hold particular significance [4].

In the solid state, the intrinsic lamellar packing of surfactants combined with the incorporation of magnetic ions presents a promising avenue for developing low-dimensional magnetic lattices (0D, 1D, or 2D) [5,6,7,8]. In such layered structures, the organic component of the metallosurfactant acts as a modulator of long-range interactions, enabling the fine-tuning of specific properties such as magnetism, conductivity, or superconductivity [9,10]. Beyond their potential applications, low-dimensional magnets provide a rich platform for exploring quantum spin phases. In many cases, strong interactions within a low-dimensional magnetic lattice (D < 3) do not necessarily lead to long-range magnetic order. Instead, they can give rise to strongly entangled exotic quantum phases, such as quantum spin liquid states [11]. Even in the case of 0D magnetic dimers, complex behaviors emerge, exemplified by the Shastry-Sutherland model—a 2D network of orthogonal interacting spin- *S* = 1/2 dimers—which exhibits a diverse range of quantum spin phases under applied magnetic fields and pressure [12,13]. Hybrid organic-inorganic (HOI) metallosurfactants offer an alternative route for synthesizing such quantum magnetic systems, further expanding the possibilities for tailored low-dimensional materials [9].

The incorporation of pyridinium-based surfactants into HOI structures offers significant advantages, as their hydrogen bonding and π-stacking capabilities can introduce a diverse range of novel properties to a material [10,14,15,16]. F. Neve et al. pioneered research on HOI ionic liquids (ILs) containing pyridinium-based surfactants and tetrahalometallate [MX_4_]^2−^ anions [10,14,17]. More recently, A. Abouserie et al. explored the correlation between thermotropic phase behavior and crystal structure in *N*-butylpyridinium, (C_4_Py)_2_[MCl_4_], and *N*-dodecylpyridinium, (C_12_Py)_2_[MCl_4_], ILs, where M = Cu, Co, or Zn [15]. Additionally, the solution properties of metallosurfactants of the type (C_*n*_Py)[MCl_4_] (*n* = 14 or 16) have been extensively investigated, further expanding their potential applications [18,19,20,21].

Beyond the organic component, selecting metal-containing building blocks is crucial in shaping the properties of HOI metallosurfactants. Incorporating iron-based species enhances their versatility and functionality, as iron can adopt multiple oxidation states and form both high-spin and low-spin complexes. Among various iron-containing HOI compounds, those featuring [Fe_2_OX_6_]^2−^ anions have garnered significant interest. Since the first structural report of (*μ*-oxo)bis[trichloroferrate(III)], [Fe_2_OCl_6_]^2−^, in 1978 by Drew, McKee, and Nelson [22], numerous related compounds incorporating the [Fe_2_Cl_6_O]^2−^ [23,24,25] and [Fe_2_Br_6_O]^2−^ [26,27] dianions have been synthesized and characterized. These species are prominent in the coordination chemistry of trivalent iron, with the Fe–O–Fe unit drawing particular attention due to its magnetic properties and its relevance to the active sites of various metalloproteins [24,25,28,29]. Moreover, high-valent metal-oxo species featuring multiple metal-oxygen bonds are recognized as key intermediates in oxidation reactions in numerous biological and synthetic catalytic cycles [30].

These considerations guided us to develop a more advanced class of metallosurfactant hybrids based on the *N*-dodecylpyridinium cation (C_12_Py)^+^. Specifically, we present a series of dodecylpyridinium metallosurfactants featuring tetrahaloferrate(III) anions, (C_12_Py)[FeX_4_], and for the first time those containing *μ*-oxo-hexahalo-diferrate(III), (C_12_Py)_2_[Fe_2_X_6_O], ions, where X = Cl or Br. A combination of techniques—including conductometry, dynamic and electrophoretic light scattering, single-crystal X-ray diffraction, thermogravimetric analysis, and magnetic measurements—was employed to investigate the effect of complex inorganic counterions on the solution and solid-state properties of the metallosurfactants. It was found that the two types of synthesized C_12_Py-metallosurfactants significantly differ in the arrangement of molecules in crystals (interdigitated vs. non-interdigitated bilayers of cations, different cation conformations), magnetic properties (paramagnetic vs. nonmagnetic singlet ground state), as well as self-assembly (vesicles vs. micelles). However, all synthesized metallosurfactants display enhanced physico-chemical properties compared to the parent surfactant. Furthermore, we find that (C_12_Py)_2_[Fe_2_X_6_O] metallosurfactants behave as antiferromagnetic dimers with singlet ground state formed by strong antiferromagnetic interaction between the two Fe spins *S* = 5/2 mediated by the *µ*-oxo bridge. Finally, as one of the synthesized compounds with the tetrahalogenoferrate anion, (C_12_Py)[FeClBr_3_], could not be isolated as a solid at room temperature, and another, (C_12_Py)[FeCl_4_], was found to have a melting point of 50 °C, we classify them as ionic liquids (ILs).

## 2. Results and Discussion

The reaction of C_12_PyCl and FeCl_3_·6H_2_O in methanol in a molar ratio 1:1 yielded (C_12_Py)[FeCl_4_] in the form of bright yellow plate-like crystals. No crystal product was isolated when the analogous reaction was conducted using FeBr_3_. Instead, a jelly-like material was left behind after the complete evaporation of the solvent. Comparison of its FT-IR spectra with that of (C_12_Py)[FeCl_4_] (Supplementary Materials Figure S1), unveiled their close resemblance. Moreover, TG/DSC analysis revealed a degradation scenario comparable to that of (C_12_Py)[FeCl_4_] (Supplementary Materials Figure S2), without melting, however. Based on that we anticipate this product is an ionic liquid of the composition (C_12_Py)[FeClBr_3_]. The difference in product state (solid vs. liquid) may be attributed to the weaker interactions between C_12_Py^+^ and [FeClBr_3_]^−^ compared to [FeCl_4_]^−^, leading to an ionic liquid at room temperature. Since (C_12_Py)[FeClBr_3_] is a room-temperature ionic liquid, it was not further investigated in this study, as the primary focus was on exploring the solid-state magnetism of C_12_Py-metallosurfactants.

Reactions of C_12_PyCl with Fe(II) salts, namely FeCl_2_·4H_2_O and FeBr_2_, were performed under an inert argon atmosphere while using dry methanol as a solvent, thus trying to preserve the lower oxidation state of iron in the products. The reaction mixtures were evaporated immediately after the reaction, yielding in both cases crystalline material. Analysis of the material unveiled that in the case of FeCl_2_·4H_2_O, (C_12_Py)_2_[Fe_2_Cl_6_O] was obtained, whereas in the case of FeBr_2_, a mixed-halide type of salt (C_12_Py)_2_[Fe_2_Cl_3_Br_3_O] formed (vide infra). Close inspection of their FT-IR spectra confirms the presence of [Fe_2_X_6_O]^2−^ (X = Cl or Br) anion in both solids, as evident by a strong band at ca 875 cm^−1^ corresponding to a Fe–O–Fe stretching (Supplementary Materials Figure S1). The remaining spectral features are analogous to those of (C_12_Py)[FeX_4_] (X = Cl, Br). Results of TG/DSC analysis unveil similar degradation scenarios upon heating. (C_12_Py)_2_[Fe_2_Cl_6_O] undergoes melting at 101 °C, while (C_12_Py)_2_[Fe_2_Cl_3_Br_3_O] melts at 97 °C (Supplementary Materials Figure S2). Afterward, both compounds undergo a two-step degradation in the ca 200 °C to 400 °C range.

### 2.1. Crystal Structures

Single crystals of (C_12_Py)[FeCl_4_], (C_12_Py)_2_[Fe_2_Cl_6_O], and (C_12_Py)_2_[Fe_2_Cl_3_Br_3_O] were obtained from methanol solutions via evaporation of the reaction mixtures. Crystals of (C_12_Py)_2_[Fe_2_Cl_3_Br_3_O] were generally of lower quality compared to those of (C_12_Py)[FeCl_4_] and (C_12_Py)_2_[Fe_2_Cl_6_O], likely due to the partial chloride/bromide substitution in the samples (It should be noted that it took at least ten synthetic and (re)crystallization attempts to obtain a crystal specimen of (C_12_Py)[Fe_2_Cl_3_Br_3_O] of sufficient quality for the X-ray diffraction experiment.). The studied crystal of (C_12_Py)[FeCl_4_] proved to be a twin, and its structure was refined as a two-component inversion twin.

**Figure 1:**
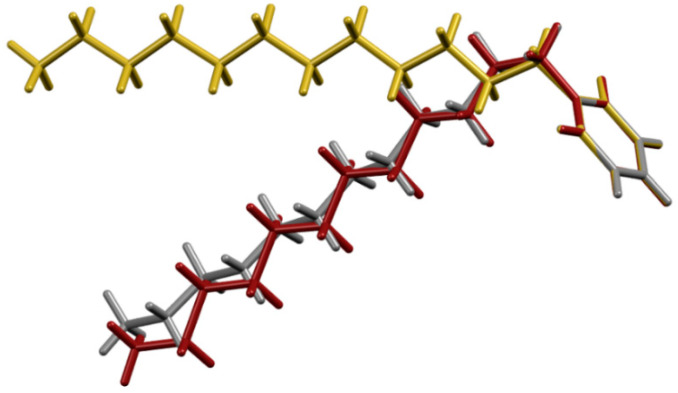
Overlay of C_12_Py^+^ conformations observed in: (C_12_Py)[FeCl_4_]—yellow, (C_12_Py)_2_[Fe_2_Cl_6_O]—gray, and (C_12_Py)_2_[Fe_2_Br_3_Cl_3_O]—dark red.

**Figure 2:**
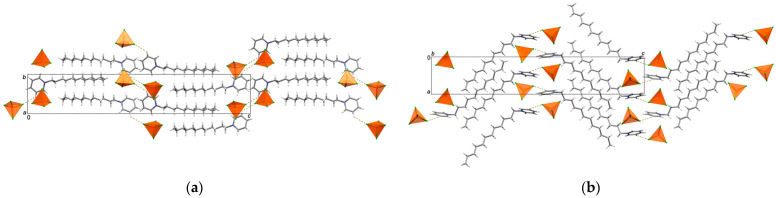
Crystal packing in (C_12_Py)[FeCl_4_] shown down the: (**a**) *a*-axis; (**b**) *b*-axis. C–H···Cl interactions are highlighted as yellow dashed lines. In both (**a**,**b**) [FeCl_4_]^−^ anions are highlighted in polyhedral style. In (**a**,**b**) the same molecules are shown.

C_12_Py^+^ cation comprises a structurally rigid pyridinium head and conformationally flexible alkyl tail. The overall conformation of the cation is dictated by the relative orientation of the tail with respect to the pyridine ring, as well as by the twisting of the alkyl tail, both evident from the relevant torsion angles. A survey of the CSD (Cambridge Structural Database) unveiled altogether nine C_12_Py^+^-containing structures, which clearly illustrate the appreciable conformational flexibility of the cation (Supplementary Materials Table S2). In the structures investigated here, significant differences in the cation conformation are observed as well (Figure 1; vide infra).

(C_12_Py)[FeCl_4_] crystallizes in the space group *P*2_1_2_1_2_1_ with one C_12_Py^+^ cation and [FeCl_4_]^−^ anion per asymmetric unit (Supplementary Materials Figure S3). The C_12_Py^+^ cation adopts a conformation where the alkyl chain is fully extended and forms an obtuse angle with respect to the plane of the pyridine ring (Figure 2). In the [FeCl_4_]^−^ the four Fe–Cl distances have slightly different values (Supplementary Materials Table S3). The [FeCl_4_]^−^ anion exhibits a nearly ideal tetrahedral geometry, as evidenced by the corresponding τ_4_ value of 0.99 (Supplementary Materials Table S3) [31]. In the crystal structure, cations and anions associate via C–H···Cl interactions, forming in this way interdigitated bilayers of cations with thickness *c*/2.

**Figure 3:**
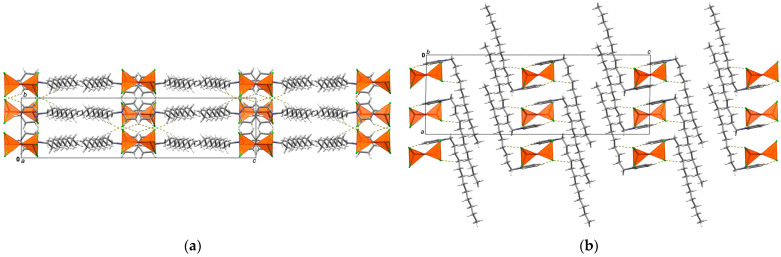
Crystal packing in (C_12_Py)_2_[Fe_2_Cl_6_O] shown down the: (**a**) *a*-axis; (**b**) *b*-axis. C–H···Cl interactions are highlighted as yellow dashed lines. In both (**a**,**b**) [Fe_2_Cl_6_O]^2−^ anions are highlighted in polyhedral style. In (**a**,**b**) the same molecules are shown.

**Figure 4:**
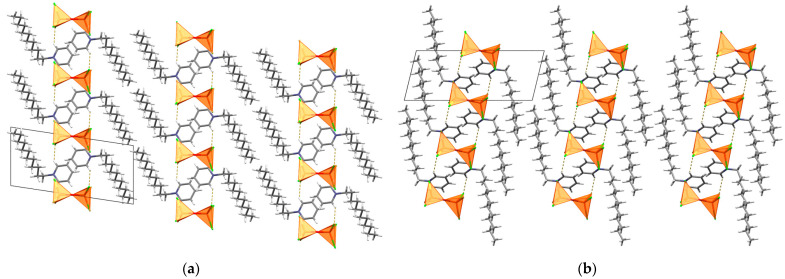
Crystal packing in (C_12_Py)_2_[Fe_2_Br_3_Cl_3_O] shown down the: (**a**) *a*-axis; (**b**) *b*-axis. C–H···Cl interactions are highlighted as yellow dashed lines. In both **(a**,**b**) [Fe_2_Br_3_Cl_3_O]^2−^ anions are highlighted in polyhedral style. In (**a**,**b**) the same molecules are shown.

[Fe_2_X_6_O]^2−^ (X = Cl or Br) is not that uncommon in the chemistry of iron(III). A survey of the CSD database unveiled overall 89 entries for [Fe_2_Cl_6_O]^2−^ containing structures, nine entries for the [Fe_2_Br_6_O]^2−^ containing structures, and none for those containing mixed halide type of [Fe_2_X_6_O]^2−^ anions [32,33]. In all inspected cases both Fe(III) atoms of the anion assume nearly ideal tetrahedral geometry. Due to the free rotation of Fe–O linkages there is an assortment of conformations that the anion can have, which can be classified into three major groups: eclipsed, skew, and staggered conformations (for a detailed description see Supplementary Materials Figure S4). Important structural features of such species, especially in the context of magnetism, are Fe–O–Fe angle, and Fe–O distances. Careful analysis of the relevant data unveils that the corresponding angle in the systems containing [Fe_2_Cl_6_O]^2−^ anions swings between 131.71(7)° (CSD refcode: PIXSUL [23]) and 180°, with over 40% of structures assuming the latter value. In the [Fe_2_Br_6_O]^2−^ containing structures this angle ranges between 141.0(4)° (CSD refcode: ESUJUA [34]) and 180°, with over 80% of them being linear. When it comes to Fe–O distances, in the group of [Fe_2_Br_6_O]^2−^ structures these range between 1.736 Å (CSD refcode: AWIDEQ [35]) and 1.775(6) Å (CSD refcode: ESUJUA [34]) values. A similar situation is observed for [Fe_2_Cl_6_O]^2−^, where the smallest distance is 1.65(2) Å (CSD refcode: ROLMUA03 [36]) and the largest 2.01(4) Å (CSD refcode: ROLMUA03 [36]).

(C_12_Py)_2_[Fe_2_Cl_6_O] crystallizes in *I*2/*a* space group with one C_12_Py^+^ cation and half of the [Fe_2_Cl_6_O]^2−^ anion per asymmetric unit, as the oxygen atom of the [Fe_2_Cl_6_O]^2−^ anion is situated on a two-fold rotation axis (Supplementary Materials Figure S3). The C_12_Py^+^ cation adopts an L-shaped conformation, where the alkyl chain forms nearly a right angle regarding the plane of the pyridine ring (Figure 3). Compared to its orientation in (C_12_Py)[FeCl_4_], there is a noticeable difference in the alignment of the alkyl tail relative to the pyridyl ring, demonstrating the structural flexibility of the C_12_Py^+^ cation (Figure 1). In the [Fe_2_Cl_6_O]^2−^ anion each Fe atom exhibits a tetrahedral coordination geometry, as indicated by the τ_4_ value being 0.99 (Supplementary Material Table S4). The three Fe–Cl distances are slightly different. The Fe–O–Fe bridge is not linear but slightly bent, as evidenced by a Fe–O–Fe angle of 175.83(12)°. Considering the spatial arrangement of the chlorine atoms coordinated to the Fe centers, the conformation of the anion can be classified as skew. There is an abundance of C–H···Cl interactions in the crystal structure, while cations form non-interdigitated bilayers with thickness *c*/2. The layer thickness in (C_12_Py)_2_[Fe_2_Cl_6_O] is smaller as compared to (C_12_Py)[FeCl_4_], which can be associated with a difference in cation conformation.

(C_12_Py)_2_[Fe_2_Br_3_Cl_3_O] crystallizes in *P*$\bar{1}$ space group with one C_12_Py^+^ cation and half of the [Fe_2_Br_3_Cl_3_O]^2−^ anion per asymmetric unit, as the oxygen atom of the [Fe_2_Br_3_Cl_3_O]^2−^ anion is situated on an inversion center (Supplementary Materials, Figure S3). Here, the C_12_Py^+^ cation adopts a very similar conformation to that observed in the (C_12_Py)_2_[Fe_2_Cl_6_O] structure (Figure 1 and Figure 4). In the [Fe_2_Br_3_Cl_3_O]^2−^ anion each Fe atom exhibits a tetrahedral coordination geometry (τ_4_ = 0.99; Supplementary Materials Table S5). The overall geometry of [Fe_2_Br_3_Cl_3_O]^2−^ anion is similar to the [Fe_2_Cl_6_O]^2−^ one, but the partial substitution of Cl by Br leads to longer Fe–Br and Fe–Cl bond lengths compared to the Fe–Cl bonds in [Fe_2_Cl_6_O]^2−^, consistent with the larger ionic radius of bromine (Supplementary Materials Table S5) [37]. Moreover, the Fe–O–Fe bridge here is linear, as the bridging oxygen atom resides on the inversion center. The conformation of the anion is best described as staggered when considering the spatial arrangement of the halogen atoms coordinated to the Fe atoms. Crystal packing relies on C–H···Cl interactions and is comparable to that in the (C_12_Py)_2_[Fe_2_Cl_6_O], characterized by bilayers of cations of the thickness *c*.

### 2.2. Magnetic Properties

**Figure 5:**
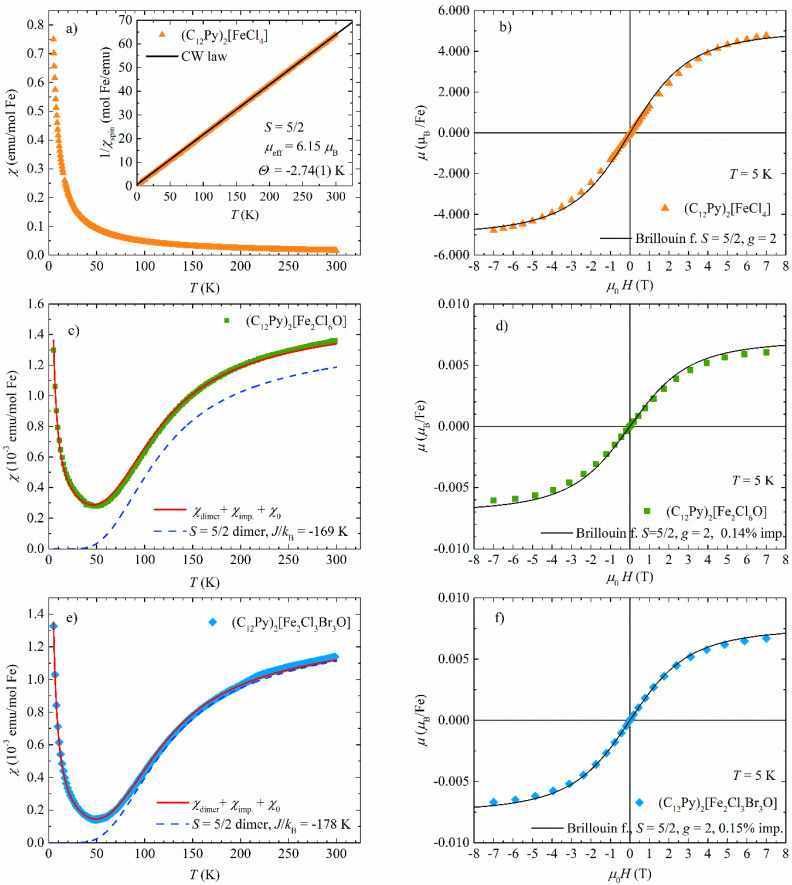
The temperature dependence of magnetic susceptibility of (**a**) (C_12_Py)_2_[FeCl_4_], (**c**) (C_12_Py)_2_[Fe_2_Cl_6_O], and (**e**) (C_12_Py)_2_[Fe_2_Cl_3_Br_3_O] measured in *µ*_0_*H* = 0.1 T. Solid black line in the inset of (**a**) represents fit to the Curie-Weiss law. The red solid line in (**c**,**e**) represents fit to Equation (1), and the dashed blue line is the result for spin *S* = 5/2 dimer only. Magnetic field dependence of magnetic moment of (**b**) (C_12_Py)_2_[FeCl_4_], (**d**) (C_12_Py)_2_[Fe_2_Cl_6_O] and (**f**) (C_12_Py)_2_[Fe_2_Cl_3_Br_3_O] measured at *T* = 5 K. Black solid line represents Brillouin function for spin *S* = 5/2 with *g* = 2 for (**b**) one spin per Fe and (**d**) 0.14% and (**f**) 0.15% of impurity spin *S* = 5/2.

The temperature dependence of magnetic susceptibility of (C_12_Py)[FeCl_4_] is shown in Figure 5a. It can be well described by the expression

(1) $$\chi \;\left(T\right)=\frac{C}{T-\Theta }+\chi_{0}$$

where the first term is Curie–Weiss law describing the paramagnetic response of the magnetic transition metal ions, and the second is the temperature-independent part of the susceptibility, *χ*_0_. In Equation (1), *C* is the Curie constant and *Θ* is the Curie–Weiss temperature. The Curie constant is given by *C* = *N*_A_ *µ*_eff_^2^/3*k*_B_ where *N*_A_ is Avogadro’s constant, *k*_B_ the Boltzmann constant, and *µ*_eff_ the effective magnetic moment. *µ*_eff_ = *gµ*_B_ [*S* (*S* + 1)]^1/2^, where *g* is the electron *g* factor, *µ*_B_ the Bohr magneton, and *S* is the spin of the transition metal ion.

The Curie–Weiss temperature *Θ*, expressed in Kelvin, corresponds to the effective magnetic interactions between the magnetic moments, where the positive value of *Θ* reflects the ferromagnetic interactions and the negative value of the antiferromagnetic interactions. The agreement of the fit of Equation (1) to the data is shown by a solid black line in the inset of Figure 5a. We obtain the effective moment *µ*_ef_ = 6.15 *µ*_B_, which agrees with the expected value for Fe^3+^ spin *S* = 5/2 [38]. The obtained value of the Curie–Weiss temperature *Θ* = −2.74(1) K signifies very weak antiferromagnetic interactions between the Fe^3+^ spins. The almost-noninteracting paramagnetic behavior is corroborated by the field dependence of the magnetic moment measured at *T* = 5 K (Figure 5b), which can be well described by the Brillouin function for the spin *S* = 5/2 and *g* = 2 (solid black line in Figure 5b).

**Figure 6:**
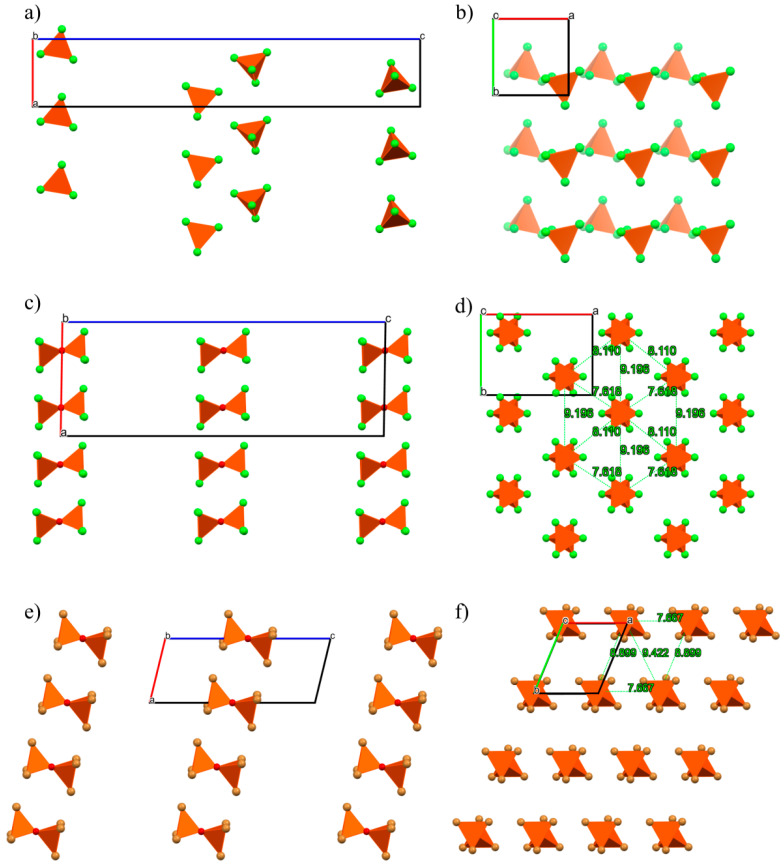
(**a**) Crystal packing in (C_12_Py)[FeCl_4_] where the separated [FeCl_4_] tetrahedra (orange) form two rows in the *ab* plane. (**b**) One magnetic double-layer in the *ab* plane where the darker tetrahedra represent the top layer and lighter the bottom layer. (**c**) Crystal packing in (C_12_Py)_2_[Fe_2_Cl_6_O] where [Fe_2_Cl_6_O]^2−^ anions (orange) form oxygen-bridged magnetic dimers, the layers of which are isolated by dodecylpyridinium cations (omitted for clarity). (**d**) [Fe_2_Cl_6_O]^2−^ magnetic dimers in (C_12_Py)_2_[Fe_2_Cl_6_O] form 2D magnetic lattice in the *ab* plane. (**e**) Crystal packing of (C_12_Py)_2_[Fe_2_Cl_3_Br_3_O]. Where [Fe_2_Cl_3_Br_3_O]^2−^ anions (orange) form oxygen-bridged magnetic dimers, the layers of which are isolated by dodecylpyridinium cations (omitted for clarity). (**f**) 2D magnetic plane of [Fe_2_Cl_3_Br_3_O]^2−^ magnetic dimers. Both Cl and Br are shown in the same brown color in (**e**,**f**), while Cl is green in (**a**–**d**).

The obtained result can be well understood if we take a look at the crystal structure of (C_12_Py)[FeCl_4_] (Figure 6a,b). One Fe^3+^ ion is surrounded by four Cl^−^ ions, forming an isolated tetrahedron. [FeCl_4_]^−^ tetrahedra (shown in orange in Figure 6a,b) are basic magnetic units in the compound and are confined to planes (Figure 6b) separated by the dodecylpyridinium cations (Figure 6a). Neighboring tetrahedra do not share Cl^−^ ions, so there is no direct superexchange between the Fe^3+^ magnetic moments, which is reflected in the almost vanishing Curie-Weiss temperature *Θ*.

The temperature dependence of the magnetic susceptibility of (C_12_Py)_2_[Fe_2_Cl_6_O] is shown in Figure 5c. The susceptibility monotonically decreases as the temperature decreases and reaches a minimum around *T* = 50 K. Below that temperature, the susceptibility increases steeply as the temperature decreases in a Curie-like manner. This behavior can be understood quantitatively when we realize that in this metallosurfactant, [Fe_2_Cl_6_O]^2−^ anions are basic magnetic units where the Fe^3+^ ion sits within a {Cl_3_O} tetrahedron while two {FeCl_3_O} tetrahedra share a single oxygen (Figure 6c,d; and Supplementary Materials Figure S3). The superexchange interaction between the Fe^3+^ spins is mediated via the bridging oxygen and is expected to be quite strong because of the almost linear Fe–O–Fe bond which favors strong antiferromagnetic interactions according to Goodenough–Kanamori–Anderson rules [39,40,41]. Detailed studies of the influence of the Fe–O–Fe angle on the strength of the superexchange interaction can be found in the literature for (*µ*-oxo)bis[trichloroferrate(III)] dianon salts [24,25]. Due to an intrinsic layered structure, dodecylpyridinium cations separate the layers containing [Fe_2_Cl_6_O]^2−^ magnetic units (Figure 6c), which form a 2D magnetic lattice of magnetic dimers (Figure 6d).

The oxygen-sharing {FeCl_3_O} tetrahedra are expected to form spin dimers where superexchange interaction is mediated through the bridging oxygen. For Fe^3+^ spin *S* = 5/2 isolated dimers, the interaction between the spins *S*_1_ and *S*_2_ in the dimer is well described by the Heisenberg Hamiltonian [42]

(2) $$H=-2J\;S_{1}\cdot S_{2}$$

where *J* denotes the intradimer superexchange interaction between the spins ***S*_1_** and ***S*_2_**. For antiferromagnetic interaction between the spins, *J* < 0 and vice versa for ferromagnetic interaction. The temperature dependence of susceptibility per spin of the AFM dimer with two spins *S*_1_ = *S*_2_ = 5/2 is given by [25,42]

(3) $$\chi_{dimer}\left(T\right)=\frac{Ng^{2}\mu_{B}^{2}}{k_{B}T}\cdot \frac{e^{2x}+5e^{6x}+14e^{12x}+30e^{20x}+55e^{30x}}{1+3e^{2x}+5e^{6x}+7e^{12x}+9e^{20x}+11e^{30x}}$$

where *x* = *J*/*k*_B_*T*, *g* is the electron *g* factor, *µ*_B_ is the Bohr magneton, *k*_B_ is the Boltzmann constant. The low-temperature upturn in susceptibility comes from unpaired spins *S* = 5/2, where a small part of Fe^3+^ ions is not paired in AFM dimers but rather represents the paramagnetic impurity contribution. The observed susceptibility data can, therefore, be described by the following expression [24,25]

(4) $$\chi \left(T\right)=(1-p)\cdot \chi_{dimer}\left(T\right)+p\cdot \chi_{imp}\left(T\right)+\chi_{0}$$

where *χ*_dimer_(T) is the susceptibility of the antiferromagnetic dimer with *S*_1_ = *S*_2_ = 5/2 (Equation (3)), *χ*_imp_(T) is the impurity contribution (Equation (5) below), and *χ*_0_ is the weak temperature-independent susceptibility. We assume that the impurity concentration *p* reflects the number of unpaired spins *S* = 5/2 from broken dimers, thus leaving (1 − *p*) spins within dimers. The Curie-like upturn of the unpaired spins *S* = 5/2 is given by

(5) $$\chi_{imp}\left(T\right)=\frac{Ng^{2}\mu_{B}^{2}S\left(S+1\right)}{3k_{B}T}$$

where we assume that there are no interactions between the unpaired spins, i.e., the Curie–Weiss temperature *Θ* is zero. This assumption is reasonable for small impurity concentrations. The fit of Equation (4) to the measured data gives *J*/*k*_B_ = −169 K, with *p* = 0.141(1)% and *χ*_0_ = 3.09(2)·10^−4^ emu/mol Fe^3+^. In Figure 5c, we plotted the fitting curve (red line) compared to the measured data. We see that the agreement is excellent. The dashed blue line in Figure 5c shows the dimer-only susceptibility (Equation (3)), which goes to zero below approx. 40 K, signifying a spin-singlet ground state. The obtained percentage of 0.14% of uncoupled (impurity) spins *S* = 5/2 obtained from the fit is responsible for the observed magnetic field dependence of magnetic moment described by the Brillouin function for *S* = 5/2 and *g* = 2 (see Figure 5d)

The temperature dependence of susceptibility of (C_12_Py)_2_[Fe_2_Cl_3_Br_3_O] is shown in Figure 5e. We observe the same behavior as for (C_12_Py)_2_[Fe_2_Cl_6_O]. Similar to (C_12_Py)_2_[Fe_2_Cl_6_O] the basic magnetic unit is formed by oxygen-sharing [Fe_2_Cl_3_Br_3_O]^2−^ magnetic dimers (Figure 6e,f). The dimers are confined to 2D planes (in *ab* plane, Figure 6e,f) separated by the dodecylpyridinium cations. Like in its chlorine counterpart, strong intradimer superexchange interaction is expected to be mediated via the common oxygen forming an effective magnetic dimer with spins *S*_1_ = *S*_2_ = 5/2.

The measured susceptibility can be well described by Equation (4) where the parameters from the fit give *J*/*k*_B_ = −178 K, *p* = 0.15%, *χ*_0_ = −2.4(5)·10^−5^ emu/mol. The small amount of unpaired spins *S* = 5/2 gives the low-T upturn in susceptibility and a field dependence at *T* = 5 K which is well described by a Brillouin function for *S* = 5/2 for impurity concentration of 0.15% per spin (see Figure 5f).

Both (C_12_Py)_2_[Fe_2_Cl_6_O] and (C_12_Py)_2_[Fe_2_Cl_3_Br_3_O] metallosurfactants have the same crystallographic motif: [Fe_2_X_6_O]^2−^ (X = Cl or Br) responsible for the magnetic properties of these compounds. The oxygen-bridged Fe^3+^ ions form antiferromagnetic dimers, which results in nonmagnetic singlet ground state. The Fe–O–Fe angles and the intradimer interaction expressed in meV and cm^−1^ are given in Table 1. Strong antiferromagnetic interaction is a consequence of almost linear, and linear Fe–O–Fe bonds in the case of (C_12_Py)_2_[Fe_2_Cl_6_O] and (C_12_Py)_2_[Fe_2_Cl_3_Br_3_O], respectively [25,39,40,41,43].

### 2.3. Self-Assembly Properties

For surfactants’ applications in solutions, emulsions, etc., understanding their self-assembly properties below and above the critical micellar concentration (cmc) is fundamental. The cmc is defined as the minimum concentration of surfactant at which micelles form and represents one of the most important physico-chemical parameter for these amphiphilic molecules.

#### 2.3.1. Solution Properties Below the cmc

**Figure 7:**
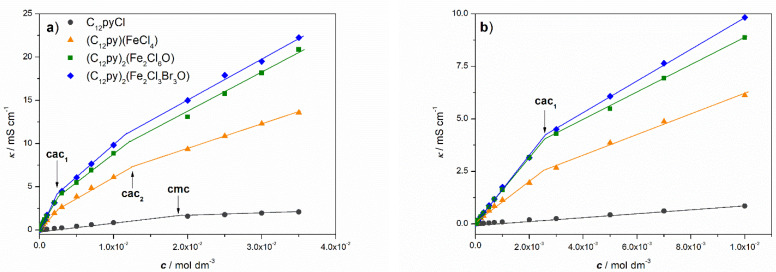
Dependence of electrical conductivity (κ) on the concentration (c) of C_12_Py-metallosurfactants and the metal-free precursor, C_12_PyCl, at 25 °C: (**a**) over the entire range of measured concentrations, and (**b**) at lower concentrations. The concentrations at which breaks in the *κ* vs. *c* curves were observed, denoted as cac_1_ and cac_2_, are indicated by arrows.

Figure 7 displays the electrical conductivity vs. concentration plots for the investigated metallosurfactants, and the metal-free precursor, shown across the full range of measured concentrations (Figure 7a) and at lower concentrations (Figure 7b). Contrary to usual, two breaks instead of one were observed on the *κ* vs. *c* curves for all C_12_Py-metallosurfactants, denoted as critical aggregation concentrations cac_1_ and cac_2_, reflecting their more complex aggregation behavior (vide infra). Slightly higher electrical conductivity values for (C_12_Py)_2_[Fe_2_Cl_3_Br_3_O] compared to (C_12_Py)_2_[Fe_2_Cl_6_O] can be attributed to the somewhat greater conductivity of Br^−^ ions compared to Cl^−^ ions [44].

**Figure 8:**
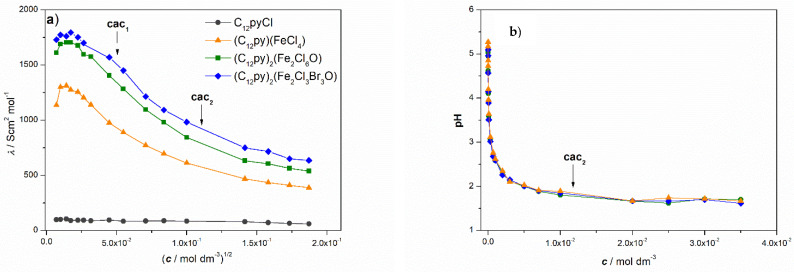
Variation of (**a**) molar conductivity (*λ*) with the square root of the concentration ($c^{1/2}$) and (**b**) pH with concentration for C_12_Py-metallosurfactants and the metal-free precursor, C_12_PyCl, at 25 °C. The concentrations at which breaks in the κ vs. c curves were observed, denoted as cac_1_ and cac_2_, are indicated by arrows.

In general, the electrical conductivity measurements are affected by interionic interactions making them a convenient method for obtaining, at the very least, qualitative indications of structural changes within surfactant solutions [45,46]. These changes, among others, include phenomena such as ion-pairing, complexation and premicellar aggregation. Therefore, the behavior of the aqueous solution of the newly synthesized C_12_Py-metallosurfactants at low concentrations was qualitatively deduced from the electrical conductivity measurements represented as the molar conductivity (*λ*) vs. the square root of the surfactant concentration ($c^{1/2}$) curves (Figure 8a).

The formation of premicellar aggregates in aqueous solutions of all investigated C_12_Py-metallosurfactants is confirmed by the shape of the *λ* vs. *c*^1/2^ curves, which display a pronounced maximum in the low concentration range *c* < cac_1_ [45,46,47]. The maximum occurs as the molar conductivity of the formed small aggregates exceeds the sum of the molar conductivities of the individual ions. For the metal-free precursor, C_12_PyCl, at *c* < cmc, no evidence of either ion pairing or premicellar aggregates was found, which is consistent with existing literature data [48].

It is well known that premicellar aggregates have a high tendency to form when the hydrophobicity of a surfactant molecule is increased, with typical examples being dimeric surfactants [45,46], including dimeric pyridinium surfactants [47]. However, this does not apply to the investigated pyridinium series of compounds. In solution, complex iron-containing counterions dissociate into various ionic species of halide and hydrated iron ions, coloring the solutions brown, and causing a significant increase in ionic strength compared to metal-free systems. In other words, the systems behave as if an electrolyte has been added, which is confirmed by the significantly higher electrical conductivity of the metallosurfactants compared to C_12_PyCl. A similar observation was reported in previously investigated metallosurfactant systems [8,49]. Heightened ionic strength results in more effective screening of the repulsive, electrostatic interactions between the quaternary ammonium pyridinium headgroups, thereby facilitating aggregation at very low concentrations.

However, the aqueous dissolution of complex counterions in question, [FeCl_4_]^−^, [Fe_2_Cl_6_O]^2−^ and [Fe_2_Cl_3_Br_3_O]^2−^, is not straightforward. When most Fe(III) salts are dissolved in water, the solution appears yellow or brown, although the [Fe(H_2_O)_6_]^3+^ ion itself is pale purple. The yellow color is due to the conjugate base [Fe(H_2_O)_5_OH]^2+^ produced by the loss of a proton. The acidic nature of Fe^3+^ ions in aqueous solutions is exacerbated by their high charge and small ionic radius, which leads to strong polarization of water molecules, facilitating proton dissociation. However, the second main species is believed to be *μ*-oxo dimer [(H_2_O)_5_FeOFe(H_2_O)_5_]^4+^ and depending on the conditions (e.g., concentration, temperature), other types of oxo species may be formed [50]. In the presence of complexing anions such as Cl^−^, the hydrolysis of Fe^3+^ is even more complex giving various chloro, aqua, and hydroxy species [50].

As shown in Figure 8b, the measured pH values decrease exponentially with increasing C_12_Py-metallosurfactant concentration, ranging from approximately 5.1 to 1.7. On the other hand, the pH of the most concentrated C_12_PyCl solution (35 mM) was 5.7. The fact that there is no significant difference in pH for all investigated metallosurfactants indicates a dominant contribution of Fe^3+^ hydrolysis in the systems, regardless of the different types of ions present.

#### 2.3.2. Aggregation Properties

As shown in Table 2, the cac_1_ values for all C_12_Py-metallosurfactants were 4–5 times lower than the cac_2_, the concentrations determined at the second break observed in the *κ* vs. *c* curves, which correspond to the cmc of C_12_PyCl. Additionally, it is important to emphasize that the cac_2_ values for the C_12_Py-metallosurfactants are lower compared to the metal-free systems, which is a trend commonly observed in the literature [4,8,18,19]. The lower cac values can be attributed to the much higher ionic strength of C_12_Py-metallosurfactants’ solutions due to the dissociation of complex inorganic counterions. Higher ionic strength promotes surfactant aggregation by screening the electrostatic repulsion between surfactant headgroups, thereby facilitating the process. The cmc for C_12_PyCl is in reasonable agreement with the previously determined values [48,51,52].

**Figure 9:**
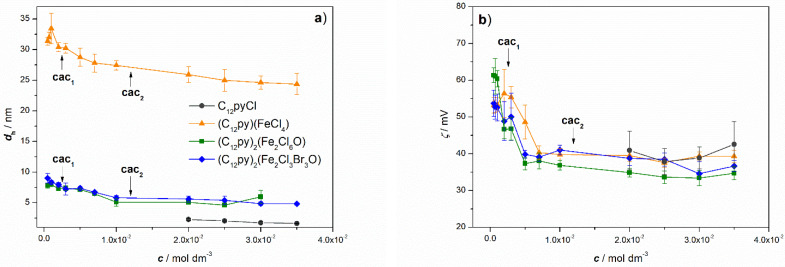
Variation of (**a**) the mean hydrodynamic diameter (*d*_h_) and (**b**) zeta potential (*ζ*) of C_12_Py-aggregates with surfactant concentration at 25 °C. The concentrations at which breaks in the *κ* vs. *c* curves were observed, denoted as cac_1_ and cac_2_, are indicated by arrows.

To determine the size and *ζ*-potential of metalloagregates as well as to confirm the formation of premicellar aggregates, DLS and ELS measurements were performed (Figure 9a,b). The existence of aggregates at *c* < cac_1_ confirmed the previous considerations and the high tendency of the investigated systems to aggregate at very low concentrations.

Consistent with existing literature, the aggregates observed in metal-free systems correspond in size to small spherical micelles (Figure 9a). Multiple studies have reported that C_12_PyCl assembles into spherical micelles within that concentration range, with a very low aggregation number (∼20), suggesting a loose packing of monomers [52,53,54]. Likewise, in both (C_12_Py)_2_[Fe_2_Cl_6_O] systems, DLS measurements confirmed the presence of small spherical micelles with diameters twice as large as those of the metal-free systems. Generally, spherical micelles of ionic surfactants gradually grow with increasing salt/electrolyte concentrations [54]. On the other hand, it is evident that in (C_12_Py)[FeCl_4_] systems, the measured aggregate sizes across the entire concentration range were significantly larger than small spherical micelles, and are much more comparable in size to small unilamellar vesicles (*d*_h_~25–30 nm) [55].

It is well-established that spherical micelles of ionic surfactants, including alkylpyridinium halides [52,56], in aqueous solutions can grow into large, cylindrical aggregates, either at high surfactant concentrations or in the presence of additives. Therefore, it would be expected that the incorporation and dissolution of complex Fe(III) counterions would induce a transition from spherical to elongated, cylindrical micelles, rather than to small vesicles. Although it is difficult to discuss the shape of aggregates based on DLS measurements, all size measurements for (C_12_Py)[FeCl_4_] systems were highly reproducible, with narrow, monomodal distributions (Supplementary Materials Figure S5). Due to the inherent limitations of the DLS technique in measuring the size of nonspherical particles, the presence of elongated, rod-like micelles would result in a bimodal size distribution, significant fluctuations, and larger standard deviations for *d*_h_. Therefore, it can be concluded that in (C_12_Py)[FeCl_4_] systems, small unilamellar vesicles are present.

For the rationalization and prediction of the types of self-assembly structures that surfactants form in a solution, the packing parameter *P* = *v*_hc_/*a*_0_ *l*_hc_ is often used, where *a*_0_, *v*_hc_, and *l*_hc_ represent the equilibrium area per molecule at the aggregate surface, the volume, and the fully extended length of the hydrophobic tail, respectively [57,58]. In other words, from a geometric perspective, the preferred surfactant aggregate structure for a given hydrophobic tail primarily depends on the effective headgroup area, *a*_0_. If electrolytes are added to the surfactant solution, the higher ionic strength screens the electrostatic repulsion between the charged surfactant headgroups. This reduces the repulsive electrostatic forces, allowing the surfactant headgroups to pack closer together and, thereby, decreasing the effective headgroup area. Moreover, at high ionic strength, dehydration of headgroups further reduces *a*_0_. Although the effective headgroup area decreases, it still accommodates the same hydrophobic volume of the surfactant tail. Consequently, the packing parameter approaches unity, which favors the formation of structures with lower curvature, such as bilayers and vesicles. Similar rationalization applies to catanionic mixtures, in which vesicles are typically formed across broad concentration ranges.

So far, the metal effect that results in micelle-vesicle transition has been mainly studied on anionic surfactants [59,60,61]. On the contrary, there are only a few reports in the literature describing the formation of vesicles in systems with alkylpyridinium compounds. S. Ghosh and J. Dey reported the formation of unilamellar vesicles in catanionic mixtures of *N*-lauroylsarcosinate with C_12_PyCl and C_16_PyCl [62]. While I. Cusano et al. reported transitions from micelles to vesicles in mixtures of C_16_PyCl and sodium diclofenac, where they identified a self-assembly transition: from spherical to linear wormlike micelles, to branched wormlike micelles, and finally, to perforated vesicles [63]. Considerably more research has shown that with the addition of relatively high concentrations of various salts, C_12_PyCl prefers to aggregate into small, spherical micelles or undergo a sphere-to-rod transition [52,53,54,56]. Therefore, it can be concluded that the presence of acidic Fe^3+^ ions and low pH in (C_12_Py)[FeCl_4_] systems significantly influence their self-assembly, leading to the formation of vesicles instead of micelles.

Why are vesicles formed only in (C_12_Py)[FeCl_4_] systems, while in both (C_12_Py)_2_[Fe_2_X_6_O] systems, as expected, only small, spherical micelles are present? The difference must be attributed to the greater neutralization of repulsive electrostatic interactions between the quaternary ammonium pyridinium headgroups in the former. Therefore, the dissociation of [Fe_2_X_6_O]^2−^ ions, with Fe–O–Fe unit, leads to the formation of ionic species that neutralize the repulsive interactions of surfactant headgroups to a lesser extent. In these systems, *μ*-oxo dimer species are present in both solution and in the solid state. Since negatively charged halide ions, Cl^−^ and Br^−^, are the ones neutralizing positively charged quaternary ammonium pyridinium headgroups, they likely have a lower concentration available in [Fe_2_X_6_O]^2−^ systems. Additionally, another factor that may contribute to the difference in the availability of free X^−^ ions in the (C_12_Py)[FeCl_4_] and (C_12_Py)_2_[Fe_2_X_6_O] systems is the differing cation-to-anion ratios, which are 1:1 and 2:1, respectively.

The degree of counterion association to the micelle/solution interface (*β*) is important in understanding the properties of surfactant micelles. The parameter *β* represents the number of counterions bound to the Stern layer of the micelles through strong electrostatic attractive interactions, reducing the micellar charge, and facilitating micelle formation. The determined *β* values (Supplementary Materials, Equation (S1)) for (C_12_Py)_2_[Fe_2_X_6_O] metallosurfactants are low compared to metal-free precursors (Table 2). This phenomenon has already been described in the literature for the tetradecylpyridinium-based metallosurfactants [18]. It has been attributed to the competitive binding of negatively charged halide counterions between the positively charged metal ion and the ionic micelles. Surprisingly, despite the lower degree of counterion association, all metallosurfactant aggregates exhibit the same or somewhat less positive *ζ*-potential at *c* < cac_2_, compared to the C_12_PyCl systems (Figure 9b).

Based on the obtained results, we can conclude that all investigated systems exhibit a high tendency to aggregate at very low concentrations, as evident from the maxima in the *λ* vs. $c^{1/2}$ curves and the presence of aggregates in DLS measurements at *c* < cac_1_. However, as shown in Figure 9a,b, a noticeable decrease in *ζ*-potential is observed at concentrations below cac_2_, as well as a less notable decrease in *d*_h_ across the entire concentration range. Moreover, measurements at lower concentrations exhibited larger standard deviations, particularly for ζ-potential values. Therefore, it is likely that less compact, irregular structures form at the lower concentrations, *c* < cac_2,_ compared to well-defined aggregates. Additionally, the decrease in *ζ*-potential and *d*_h_ with concentration is likely due to the increasing ionic strength of the solution, which reduces the thickness of the electrical double layer around the aggregates. This, in turn, leads to greater screening of the surface charge on one hand and a smaller hydrodynamic diameter on the other.

## 3. Materials and Methods

### 3.1. Materials

1-Dodecylpyridinium chloride (C_12_PyCl, 98%, Merck, Darmstadt, Germany), iron(II) chloride tetrahydrate (puriss p.a., Sigma-Aldrich, St. Louis, MO, USA), iron(III) chloride hexahydrate (puriss p.a., Sigma-Aldrich), iron(II) bromide (anhydrous, 98+%, Thermo Scientific Chemicals, Karlsruhe, Germany) and iron(III) bromide (anhydrous, 98+%, Thermo Scientific Chemicals, Karlsruhe, Germany) were used for synthesis of C_12_Py-metallosurfactants. For synthesis and purification absolute ethanol (Honeywell Riedel-de Haën, Seelze, Germany) and methanol (Kemika, Zagreb, Croatia) were used as solvents.

### 3.2. Synthesis of C_12_Py-Metallosurfactants

All C_12_Py-metallosurfactants were prepared in high yield (>90%) from a reaction involving 1-dodecylpyridinium chloride (C_12_PyCl) and divalent (FeCl_2_∙4H_2_O, FeBr_2_) or trivalent (FeCl_3_∙6H_2_O, FeBr_3_) iron halides in 1:1 molar ratio. For each synthesis approximately, 0.5 mmol of C_12_PyCl and 0.5 mmol of iron halide were dissolved and refluxed in 10 mL of methanol for 2–3 h. Synthesis involving iron(II) salts was carried out in an argon atmosphere. The solvent was evaporated using a rotary evaporator and metal complexes were isolated as brownish crystals. Except for ionic liquid (C_12_Py)[FeClBr_3_], before further analyses, synthesized compounds were repeatedly recrystallized from methanol. Purity of the samples was checked with elemental analysis: (C_12_Py)[FeCl_4_], brown crystals, C_17_H_30_NFeCl_4_, found (calculated): %C 43.99 (45.75); %H 7.11 (6.78), %N 3.08 (3.08), (C_12_Py)_2_[Fe_2_Cl_6_O], brown-gold crystals, C_34_H_60_N_2_OFe_2_Cl_6_, found (calculated): %C 49.14 (48.77); %H 7.73 (7.16), %N 3.46 (3.34), (C_12_Py)_2_[Fe_2_Cl_3_Br_3_O], brown-bronze crystals, C_34_H_60_N_2_OFe_2_Br_3_Cl_3_, found (calculated): %C 42.37 (42.07); %H 6.56 (6.23), %N 2.96 (2.89).

Synthesis of (C_12_Py)[FeX_4_]:

IR (ATR, cm^−1^). (C_12_Py)[FeCl_4_]: 2918 *υ*_a_(CH_2_); 2851 *υ*_s_(CH_2_); 1635 *υ*(py); 1484 δ(CH_3_); 1467 *δ*(CH_2_).

(C_12_Py)[FeClBr_3_]: 2923 *υ*_a_(CH_2_); 2853 *υ*_s_(CH_2_); 1634 *υ*(py); 1486 *δ*(CH_3_); 1466 *δ*(CH_2_).

Synthesis of (C_12_Py)[Fe_2_X_6_O]:

(C_12_Py)_2_[Fe_2_Cl_6_O]: 2919 *υ*_a_(CH_2_); 2850 *υ*_s_(CH_2_); 1634 *υ*(py); 1484 *δ*(CH_3_); 1464 *δ*(CH_2_); 875 *υ*_s_(Fe–O–Fe).

(C_12_Py)_2_[Fe_2_Cl_3_Br_3_O]: 2919 *υ*_a_(CH_2_); 2850 *υ*_s_(CH_2_); 1634 *υ*(py); 1484 *δ*(CH_3_); 1464 *δ*(CH_2_); 873 *υ*_s_(Fe–O–Fe).

### 3.3. Methods

Single crystal analysis. Single crystals of (C_12_Py)[FeCl_4_], (C_12_Py)_2_[Fe_2_Cl_6_O], and (C_12_Py)_2_[Fe_2_Cl_3_Br_3_O] suitable for single-crystal X-ray analysis were grown from methanol solutions. Diffraction data were collected on a Rigaku XtaLAB Synergy diffractometer equipped with a Dualflex source (Cu Kα radiation, λ = 1.54184 Å) and a HyPix detector using ω-scans. Measurements were performed at 170 K for (C_12_Py)[Fe_2_Cl_6_O] and (C_12_Py)[Fe_2_Cl_3_Br_3_O] and at 100 K for (C_12_Py)[FeCl_4_]. Data were processed with the CrysAlis software Version 1.171.43.105a suite [64]. General crystallographic parameters are summarized in Table S1 (Supplementary Materials). Crystallographic data have been deposited in the Cambridge Structural Database (CCDC deposition numbers: 2419382, 2419384, 2419385).

The structures were solved using dual space methods using SHELXT [65]. The refinement procedure was performed by full-matrix least-squares methods based on *F*^2^ values against all reflections, including anisotropic displacement parameters for all non-H atoms. Hydrogen atoms bound to carbon atoms were placed in geometrically idealized positions and refined using a riding model with *U*_iso_ = 1.2*U*_eq_ of the connected carbon atom or as ideal CH_3_ groups with *U*_iso_ = 1.5*U*_eq_. All refinements were performed using SHELXL [66]. The SHELX programs operated within the Olex2 suite [67]. Geometrical calculations were performed by Platon [68] and molecular graphics were conducted with Mercury [69]. (C_12_Py)[FeCl_4_] structure was treated as an inversion twin (two twin components are present in a 0.65: 0.35 ratio). In the (C_12_Py)[Fe_2_Cl_3_Br_3_O] structure the Cl:Br ratio was set according to elemental analysis results while constraining the atoms’ coordinates using the EXYZ flag. (C_12_Py)[Fe_2_Cl_3_Br_3_O] structure was solved as a non-merohedral two-component twin, with the two components being present in the 0.53:0.47 ratio. The reflection file contains single reflections of the first twin domain and of the second twin domain, respectively, plus the overlapping reflections. The twin law was such that the second twin-component was rotated by 180° around [1 1 0] twin axis (direct space).

FTIR ATR (attenuated total reflectance) spectra were recorded on a Nicolet iS50 spectrometer in a 4000–400 cm^−1^ spectral range

Thermogravimetric analyses were conducted on a Mettler Toledo TGA/DSC 3+ thermobalance with aluminium crucibles under a dynamic nitrogen stream of 50 mL min^−1^ flow. Experiments were conducted in a temperature range between 25 °C and 600 °C with a heating rate of 10 °C·min^−1^. The results of the experiments were processed with the Mettler Toledo STARe Evaluation Software 16.10.

Magnetic measurements. The magnetic properties of C_12_Py-metallosurfactants were studied using the Quantum Design (QD) MPMS3 VSM-SQUID magnetometer in temperatures ranging from 5 K to 300 K and in magnetic fields of up to ±7 T. The powder samples were mounted inside the standard QD VSM capsule and inserted into the QD brass sample holder. The signal from the empty capsules was also measured separately and subtracted from the total signal of the samples plus the capsule.

Preparation of solutions. All solutions were prepared in MilliQ water using volumetric flasks and were thermostated at 25 °C prior to measurement.

Electrical conductivity measurements. The electrical conductivity (κ) measurements were performed with a Conductivity Meter (Metrohm, Herisau, Switzerland) in a temperature-controlled double-walled glass container with a circulation of water. All measurements for were conducted at 25 °C.

Light scattering and zeta potential measurements. The size distribution, i.e., hydrodynamic diameter (*d*_h_), and zeta potential (*ζ*) of C_12_Py-metalloaggregates were determined by means of dynamic (DLS) and electrophoretic light scattering (ELS) techniques using a photon correlator spectrophotometer with a 532 nm green laser (Zetasizer Nano ZS, Malvern Instruments, Malvern, UK). To prevent overestimation due to the scattering of larger particles, *d*_h_ was determined as the value at the peak maximum of the volume size distributions. The reported results represent the average of six measurements. The data processing was performed using the Zetasizer software 7.13 (Malvern Instruments).

## 4. Conclusions

A series of new inorganic-organic hybrid materials—pyridinium-based metallosurfactants—was synthesized and investigated. It was established that the use of either divalent or trivalent iron halides in a straightforward synthetic procedure can yield C_12_Py-metallosurfactants with different types of complex counterions. Namely, the use of FeX_3_ type of salts (X = Cl or Br) as starting materials led to the isolation of metallosurfactants of the general formula (C_12_Py)[FeX_4_], which can be classified as ionic liquids due to their low melting points. In contrast, when FeX_2_ salts (X = Cl or Br) were used, compounds of the (C_12_Py)_2_[Fe_2_X_6_O] type formed. To the best of our knowledge, this is the first report on the synthesis of metallosurfactants containing the *μ*-oxo-hexahalo-diferrate(III) anion, as well as their self-assembly.

A combination of techniques was used to investigate newly synthesized compounds, in specific conductometry, dynamic and electrophoretic light scattering, single crystal and thermogravimetric analysis as well as magnetic measurements. All synthesized metallosurfactants display enhanced physico-chemical properties compared to the metal-free precursor. However, it was found that the incorporation of different types of iron-based counterion significantly influences the crystal structure, magnetic properties, and self-assembly behavior of the dodecylpyridinium series. Both metallosurfactants of the type (C_12_Py)_2_[Fe_2_X_6_O], as expected, self-assemble into small spherical micelles, while small vesicles were detected across the entire concentration range for (C_12_Py)[FeCl_4_]. Likewise, in crystals, differences in the arrangement of dodecyl chains and the conformation of the cations were observed.

Metallosurfactants with different counterions, (C_12_Py)[FeCl_4_] and (C_12_Py)_2_[Fe_2_X_6_O] (X = Cl, Br), show very different magnetic properties connected to the basic magnetic units present, confirming them as a fruitful platform for the design of low-D magnetic materials. Isolated [FeCl_4_]^−^ tetrahedra in (C_12_Py)[FeCl_4_] give purely paramagnetic response, while in [Fe_2_Cl_6_O]^2−^ and [Fe_2_Cl_3_Br_3_O]^2−^ strong oxygen-mediated antiferromagnetic superexchange between two spins *S* = 5/2 results in formation of 0D antiferromagnetic dimer with nonmagnetic singlet (*S*_tot_ = 0) ground state. (C_12_Py)_2_[Fe_2_Cl_6_O] and (C_12_Py)_2_[Fe_2_Cl_3_Br_3_O] therefore represent a new addition to *µ*-oxo bridged ferrate(III) dianion hybrid organic-inorganic materials with linear or almost linear Fe–O–Fe bond. In all three magnetic compounds, magnetic units form 2D planes well separated by (C_12_Py)^+^ cations, opening up possibilities for fine-tuning of long-range magnetic order, and the existence of more exotic phases to be found under extreme conditions of low temperature, high magnetic fields, and high (chemical) pressure.

## Figures and Tables

**Table 1:** The Fe–O–Fe angle and the intradimer superexchange magnitude *J* obtained from the fit of the data to Equation (1) expressed in meV and cm^−1^ compared for (C_12_Py)_2_[Fe_2_Cl_6_O] and (C_12_Py)_2_[Fe_2_Cl_3_Br_3_O] metallosurfactants. Negative sign indicates antiferromagnetic interaction.

	(C_12_Py)_2_[Fe_2_Cl_6_O]	(C_12_Py)_2_[Fe_2_Cl_3_Br_3_O]
∠(Fe–O–Fe)	175.8°	180°
*J* (meV)	−14.6	−15.4
*J* (cm^−1^)	−117.5	−123.6

**Table 2:** The critical aggregation concentrations obtained from the two breaks observed in the *κ* vs. *c* curves (cac_1_ and cac_2_) and degree of counterion binding (*β*) * for C_12_Py-metallosurfactants and metal-free precursor, C_12_PyCl, at 25 °C.

Compound	cac_1_/mM	cac_2_/mM	*β*
C_12_PyCl	-	18	0.60
(C_12_Py)[FeCl_4_]	2.6	12	-
(C_12_Py)_2_[Fe_2_Cl_6_O]	2.7	11	0.39
(C_12_Py)_2_[Fe_2_Cl_3_Br_3_O]	2.6	12	0.39

* To calculate *β*, the ratio of the slopes above and below the cac_2_ was used (Supplementary Materials, Equation (S1)).

## Data Availability

Data is contained within the article and Supplementary Material.

## Supplementary Materials

The following supporting information can be downloaded at: https://www.mdpi.com/article/10.3390/ijms26062540/s1. References [31,70] are cited in the Supplementary Materials.
