# Supplementary material for: Tuning the Properties of Dodecylpyridinium Metallosurfactants: The Role of Iron-Based Counterions
Source: Int J Mol Sci. 2025 Mar 12;26(6):2540. doi: 10.3390/ijms26062540 (PMC11942540; doi:10.3390/ijms26062540)
Supplement: Supplementary file 1 [file ijms-26-02540-s001.zip › ijms-3473154-supplementary.pdf]

Supplementary Material for:

Tuning the Properties of Dodecylpyridinium Metallosurfactants: The Role of Iron-Based Counterions

Mirta Rubčić[1], Mirta Herak[2], Ana Ivančić[3], Edi Topić[1], Emma Beriša[4], Ivana Tartaro Bujak[5], Darija Domazet Jurašin[3]*

[1] Department of Chemistry, Faculty of Science, University of Zagreb, Horvatovac 102a, HR-10000 Zagreb, Croatia; e-mail: mirta@chem.pmf.hr, edi.topic@chem.pmf.hr
[2] Department for Research of Materials under Extreme Conditions, Institute of Physics, Bijenička cesta 46, HR-10000 Zagreb, Croatia; e-mail: mirta@ifs.hr
[3] Division of Physical Chemistry, Ruđer Bošković Institute, Bijenička 54, HR-10000 Zagreb, Croatia; e-mail: darija.jurasin@irb.hr, aivancic@irb.hr
[4] Faculty of Chemical Engineering and Technology, University of Zagreb, Trg Marka Marulića 19, HR-10000 Zagreb, Croatia, e-mail: eberisa@fkit.hr
[5] Division of Materials Chemistry, Ruđer Bošković Institute, Bijenička 54, HR-10000 Zagreb, Croatia; e-mail: itartaro@irb.hr

*corresponding author:
Darija Domazet Jurašin
Division of Physical Chemistry
Ruđer Bošković Institute
Bijenička cesta 54
10 000 Zagreb, Croatia
tel: + 385 1 4561074
+ 385 98 561182
e-mail: darija.jurasin@irb.hr

Table of Contents:

Table S1. Crystal data and structure refinement for $(C_{12}Py)[FeCl_4]$, $(C_{12}Py)_2[Fe_2Cl_6O]$, and $(C_{12}Py)_2[Fe_2Cl_3Br_3O]$ 3

Figure S1. FT-IR spectra of: (a) $(C_{12}Py)[FeCl_4]$; (b) $(C_{12}Py)[FeBr_4]$; (c) $(C_{12}Py)_2[Fe_2Cl_6O]$, and (d) $(C_{12}Py)_2[Fe_2Cl_3Br_3O]$ 4

Figure S2. TGA/DSC curves for: (a) $(C_{12}Py)[FeCl_4]$; (b) $(C_{12}Py)[FeClBr_3]$; (c) $(C_{12}Py)_2[Fe_2Cl_6O]$, and (d) $(C_{12}Py)_2[Fe_2Cl_3Br_3O]$ 6

Table S2. Conformations observed in $C_{12}Py^+$ containing structures thus far reported in the literature 8

Figure S3. Molecular structures of: (a) $(C_{12}Py)[FeCl_4]$; (b) $(C_{12}Py)_2[Fe_2OCl_6]$; (c) $(C_{12}Py)_2[Fe_2OCl_3Br_3]$. In (c) the halogen positions are equally populated by Cl and Br (50:50 ratio). In: (b) *i* = -1/2-x,y,1-z; in (c) *i* = 2-x,2-y,1-z. 12

Table S3. Selected bond lengths, angles and hydrogen bond parameters in the crystal structure of $(C_{12}Py)[FeCl_4]$. 13

Table S4. Selected bond lengths, angles and hydrogen bond parameters in the crystal structure of $(C_{12}Py)_2[Fe_2Cl_6O]$. 13

Table S5. Selected bond lengths, angles and hydrogen bond parameters in the crystal structure of $(C_{12}Py)_2[Fe_2Cl_3Br_3O]$. 14

Figure S4. Illustrative examples of conformations observed in of conformations observed in a $[Fe_2X_6O]^{2-}$ containing structures (X = Cl, Br). 15

Figure S5. Measured volume size distributions in $(C_{12}Py)[FeCl_4]$ systems at different concentrations. 16

Table S1. Crystal data and structure refinement for $(C_{12}Py)[FeCl_4]$, $(C_{12}Py)_2[Fe_2Cl_6O]$, and $(C_{12}Py)_2[Fe_2Cl_3Br_3O]$.

Identification code	$(C_{12}Py)[FeCl_4]$	$(C_{12}Py)_2[Fe_2Cl_6O]$	$(C_{12}Py)_2[Fe_2Cl_3Br_3O]$
Empirical formula	$C_{17}H_{30}Cl_4FeN$	$C_{34}H_{60}Cl_6Fe_2N_2O$	$C_{34}H_{60}Br_3Cl_3Fe_2N_2O$
Formula weight	446.07	837.24	970.62
Temperature/K	170(2)	100.00(10)	170.0(1)
Crystal system	orthorhombic	monoclinic	triclinic
Space group	$P2_12_12_1$	$I2/a$	P-1
a/Å	7.2798(2)	12.7410(2)	7.6671(5)
b/Å	7.3765(2)	9.1959(2)	8.8985(6)
c/Å	41.7269(13)	36.1159(5)	17.9402(10)
α/°	90	90	93.799(5)
β/°	90	90.8820(10)	101.280(5)
γ/°	90	90	111.129(7)
Volume/Å^3	2240.71(11)	4231.02(13)	1107.04(14)
Z	4	4	1
ρ_{calc}g/cm^3	1.322	1.314	1.456
μ/mm^{-1}	1.149	9.188	10.286
F(000)	932.0	1760.0	494.0
Crystal size/mm^3	0.04 × 0.03 × 0.01	0.22 × 0.06 × 0.02	0.04 × 0.02 × 0.01
Radiation	MoKα (λ = 0.71073)	CuKα (λ = 1.54184)	
2Θ range for data collection/°	5.608 to 62.622	9.798 to 133.996	5.08 to 162.198
Index ranges	$-10 \le h \le 10$, $-10 \le k \le 10$, $-59 \le l \le 60$	$-15 \le h \le 14$, $-10 \le k \le 9$, $-43 \le l \le 43$	$-9 \le h \le 9$, $-11 \le k \le 11$, $-22 \le l \le 22$
Reflections collected	42342	10667	8535
Independent reflections	6519 [R_{int} = 0.0512, R_{sigma} = 0.0342]	3647 [R_{int} = 0.0256, R_{sigma} = 0.0291]	8535 [R_{sigma} = 0.0273]
Data/restraints/parameters	6519/0/210	3647/0/206	8535/0/208
Goodness-of-fit^a on F^2	1.049	1.047	1.062
Final R indexes^b [I>=2σ (I)]	R_1 = 0.0334, wR_2 = 0.0708	R_1 = 0.0284, wR_2 = 0.0745	R_1 = 0.0915, wR_2 = 0.2523
Final R indexes [all data]	R_1 = 0.0441, wR_2 = 0.0738	R_1 = 0.0317, wR_2 = 0.0760	R_1 = 0.1236, wR_2 = 0.2953
Largest diff. peak/hole / e Å^{-3}	0.40/-0.26	0.51/-0.30	1.68/-1.39
Flack parameter	0.354(16)	-	-

$^a S = \{\Sigma[w(F_o^2 - F_c^2)2]/(N_r - N_p)\}^{1/2}$ where N_r = number of independent reflections, N_p = number of refined parameters.

$^b R = \Sigma||F_o| - |F_c|/\Sigma|F_o|$; $wR = \{\Sigma[w(F_o^2 - F_c^2)2]/\Sigma[w(F_o^2)2]\}^{1/2}$

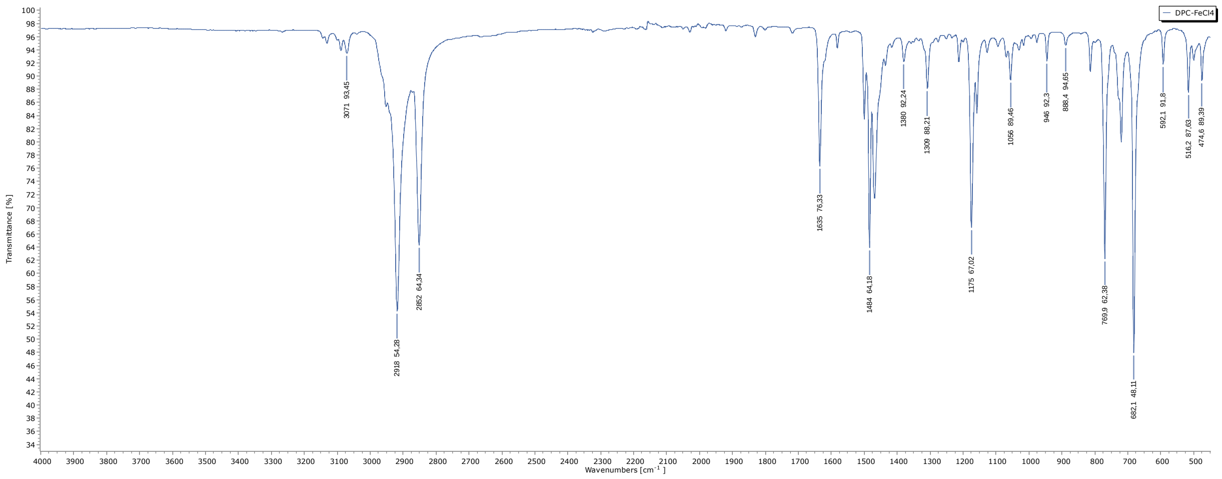
DPC-FeCl4
Transmittance [%]
Wavenumbers [cm-1]
3071 93,45
2918 54,28
2852 64,34
1635 76,33
1484 64,18
1380 92,24
1309 88,21
1175 67,02
1056 89,46
946 92,3
888,4 94,65
769,9 62,38
682,1 48,11
592,1 91,8
516,2 87,63
474,6 89,39

(a)

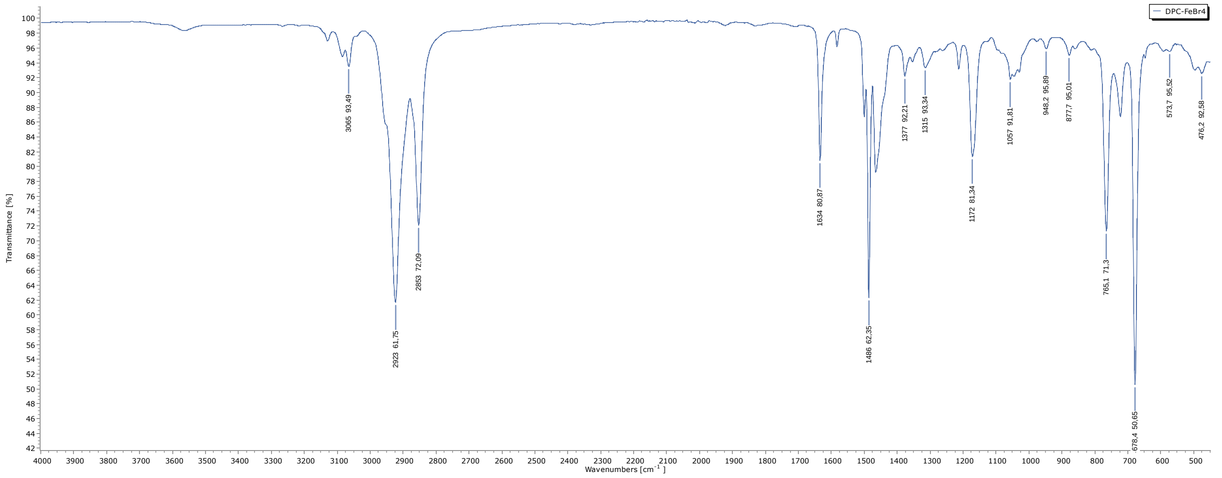
DPC-FeBr4
Transmittance [%]
Wavenumbers [cm-1]
3065 93,49
2923 61,75
2853 72,09
1634 80,87
1486 62,35
1377 92,21
1315 93,34
1172 81,34
1057 91,81
948,2 95,89
877,7 95,01
765,1 71,3
678,4 50,65
573,7 95,52
476,2 92,58

(b)

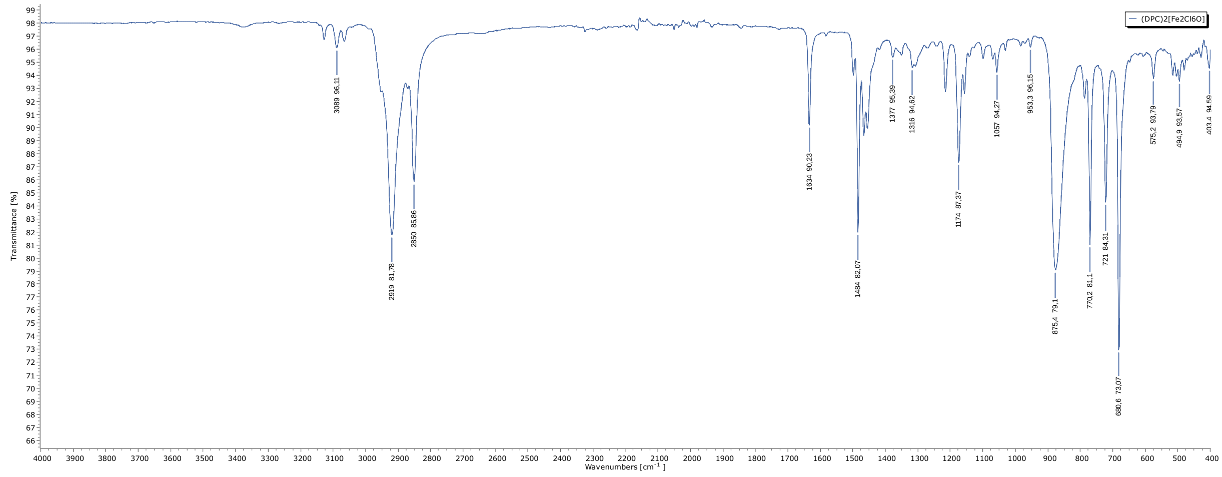
(DPC)2[Fe2Cl6O]
Transmittance [%]
Wavenumbers [cm-1]
3089 96,11
2919 81,78
2850 85,86
1634 90,23
1484 82,07
1377 95,39
1316 94,62
1174 87,37
1057 94,27
953,3 96,15
875,4 79,1
770,2 81,1
721 84,31
680,6 73,07
575,2 93,79
494,9 93,57
403,4 94,59

(c)

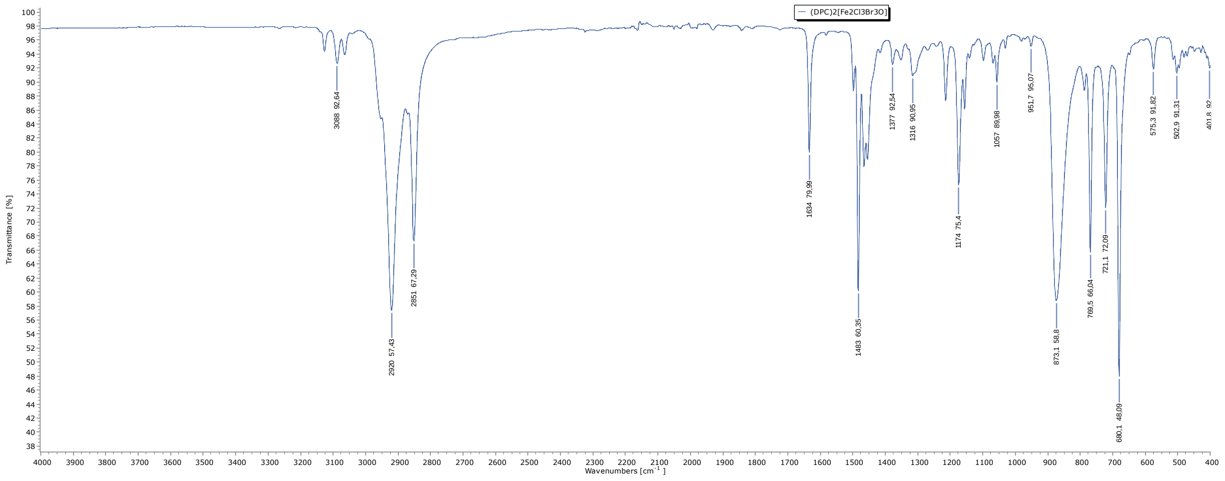

(d)

Figure S1. FT-IR spectra of: (a) $(C_{12}Py)[FeCl_4]$; (b) $(C_{12}Py)[FeBr_4]$; (c) $(C_{12}Py)_2[Fe_2Cl_6O]$, and (d) $(C_{12}Py)_2[Fe_2Cl_3Br_3O]$.

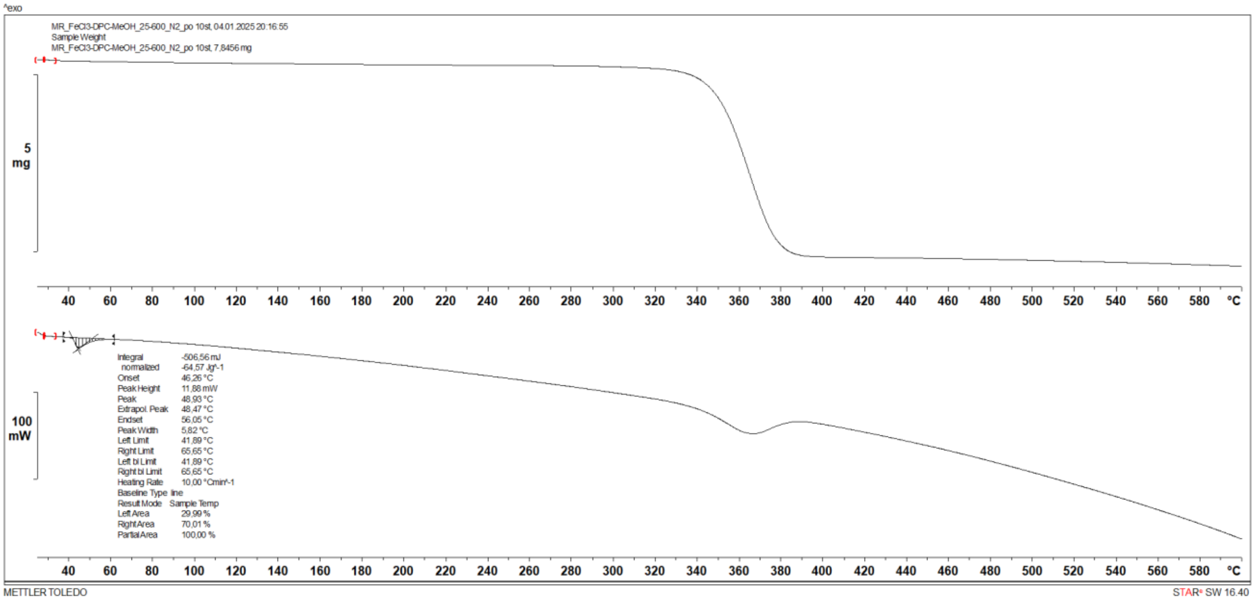
^exo
MR_FeCl3-DPC-MeOH_25-600_N2_po 10st, 04.01.2025 20:16:55
Sample Weight
MR_FeCl3-DPC-MeOH_25-600_N2_po 10st, 7,8456 mg
5
mg
40 60 80 100 120 140 160 180 200 220 240 260 280 300 320 340 360 380 400 420 440 460 480 500 520 540 560 580 °C
Integral -506,56 mJ
normalized -64,57 Jg^-1
Onset 46,26 °C
Peak Height 11,88 mW
Peak 48,93 °C
Extrapol. Peak 48,47 °C
Endset 56,05 °C
Peak Width 5,82 °C
Left Limit 41,89 °C
Right Limit 65,65 °C
Left bl Limit 41,89 °C
Right bl Limit 65,65 °C
Heating Rate 10,00 °Cmin^-1
Baseline Type line
Result Mode Sample Temp
Left Area 29,99 %
Right Area 70,01 %
Partial Area 100,00 %
100
mW
40 60 80 100 120 140 160 180 200 220 240 260 280 300 320 340 360 380 400 420 440 460 480 500 520 540 560 580 °C
METTLER TOLEDO
STAR® SW 16.40

(a)

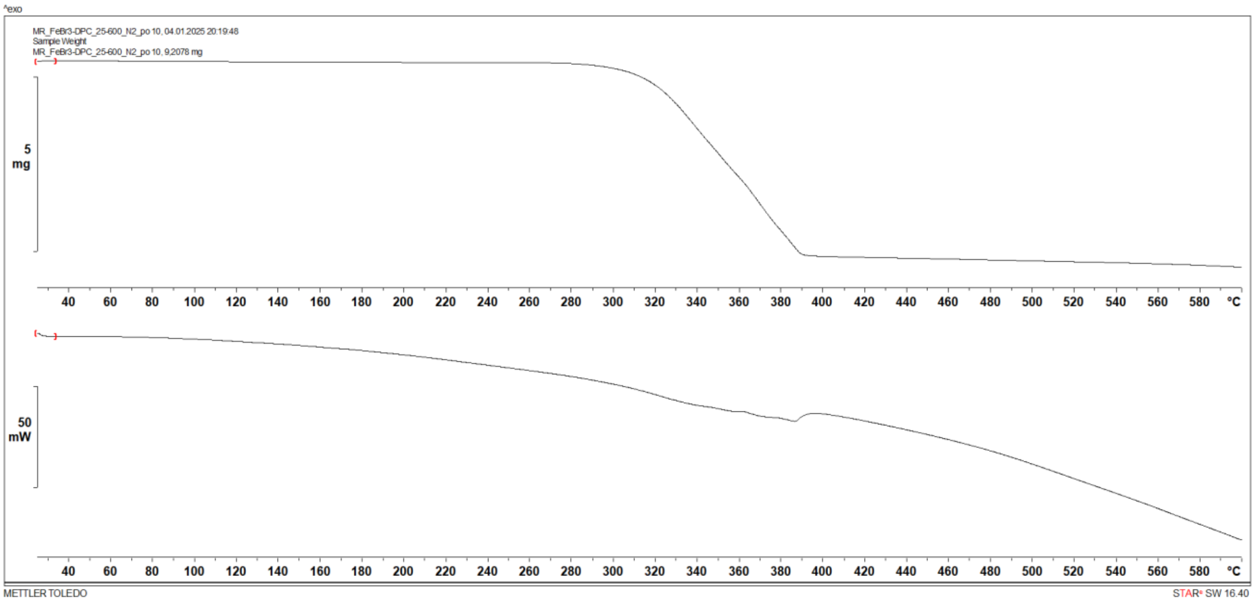
^exo
MR_FeBr3-DPC_25-600_N2_po 10, 04.01.2025 20:19:48
Sample Weight
MR_FeBr3-DPC_25-600_N2_po 10, 9,2078 mg
5
mg
40 60 80 100 120 140 160 180 200 220 240 260 280 300 320 340 360 380 400 420 440 460 480 500 520 540 560 580 °C
50
mW
40 60 80 100 120 140 160 180 200 220 240 260 280 300 320 340 360 380 400 420 440 460 480 500 520 540 560 580 °C
METTLER TOLEDO
STAR® SW 16.40

(b)

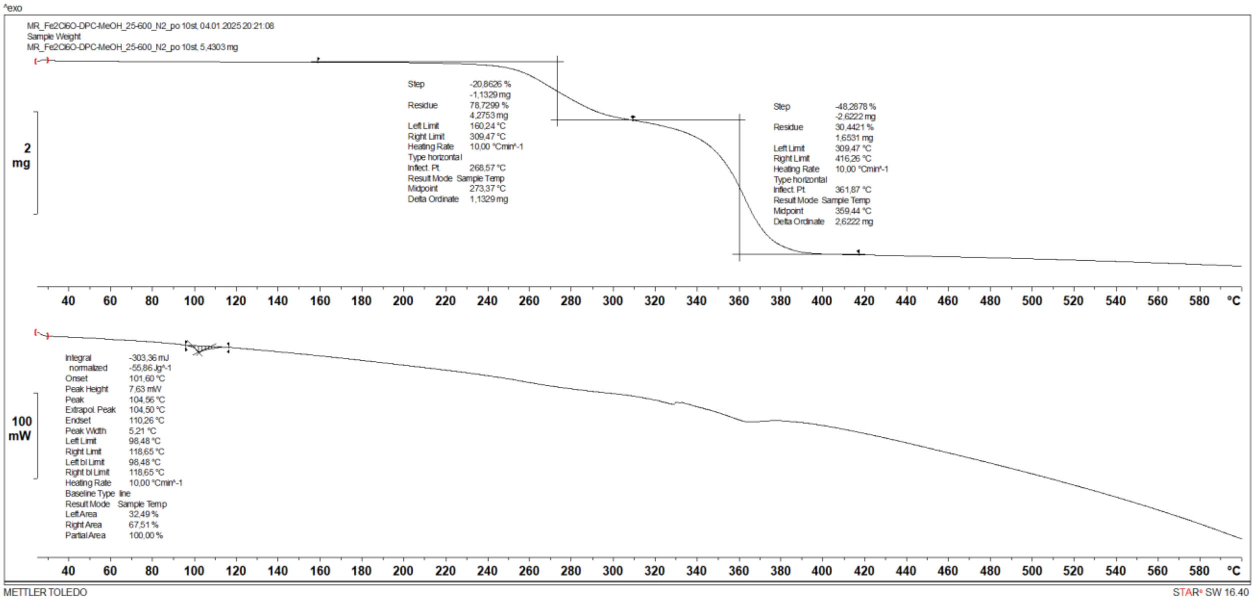

(c)

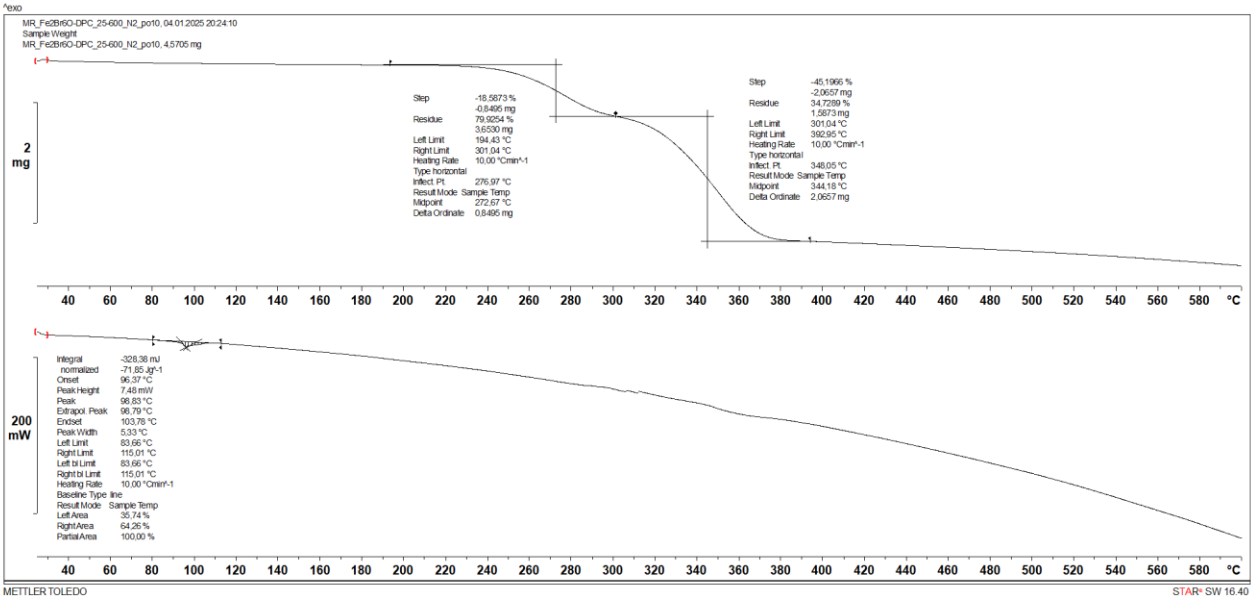

(d)

Figure S2. TGA/DSC curves for: (a) $(C_{12}Py)[FeCl_4]$; (b) $(C_{12}Py)[FeClBr_3]$; (c) $(C_{12}Py)_2[Fe_2Cl_6O]$, and (d) $(C_{12}Py)_2[Fe_2Cl_3Br_3O]$.

Table S2. Conformations observed in $C_{12}Py^{+}$ containing structures thus far reported in the literature.

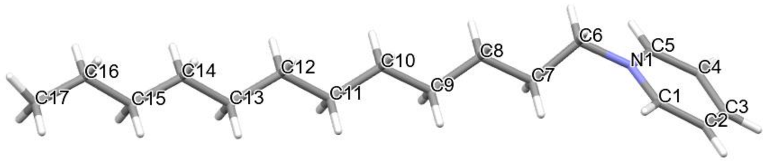

CSD refcode: FAKQUD

Atom 1	Atom 2	Atom 3	Atom 4	Torsion /°
C5	N1	C6	C7	103.8(8)
C1	N1	C6	C7	-77.5(8)
N1	C6	C7	C8	177.3(7)
C6	C7	C8	C9	-176.1(8)
C7	C8	C9	C10	178.6(9)
C8	C9	C10	C11	-178.5(9)
C9	C10	C11	C12	-179(1)
C10	C11	C12	C13	-178(1)
C11	C12	C13	C14	-179(1)
C12	C13	C14	C15	-176(1)
C13	C14	C15	C16	-180(1)
C14	C15	C16	C17	-178(1)

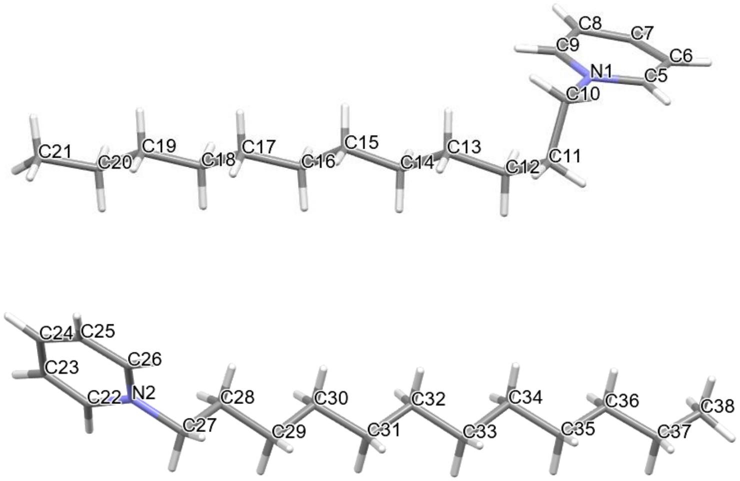

CSD refcode: FARSEZ

Atom 1	Atom 2	Atom 3	Atom 4	Torsion /°
C22	N2	C27	C28	86(2)
C26	N2	C27	C28	-96(2)
N2	C27	C28	C29	-177(2)
C27	C28	C29	C30	-173(2)
C28	C29	C30	C31	176(2)
C29	C30	C31	C32	-174(2)
C30	C31	C32	C33	178(2)
C31	C32	C33	C34	174(2)
C32	C33	C34	C35	176(2)
C33	C34	C35	C36	-178(2)
C34	C35	C36	C37	-176(3)
C35	C36	C37	C38	-176(3)
C9	N1	C10	C11	92(2)
C5	N1	C10	C11	-85(3)
N1	C10	C11	C12	-61(2)
C10	C11	C12	C13	-74(2)
C11	C12	C13	C14	-175(2)
C12	C13	C14	C15	-177(2)
C13	C14	C15	C16	-176(2)
C14	C15	C16	C17	175(2)

C15	C16	C17	C18	173(3)
C16	C17	C18	C19	-179(3)
C17	C18	C19	C20	-175(3)
C18	C19	C20	C21	176(3)

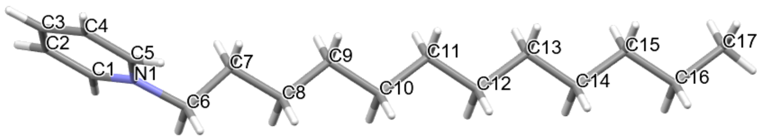

CSD refcode: OWIPES

Atom 1	Atom 2	Atom 3	Atom 4	Torsion/°
C5	N1	C6	C7	-78.2(6)
C1	N1	C6	C7	100.4(6)
N1	C6	C7	C8	179.8(4)
C6	C7	C8	C9	179.3(5)
C7	C8	C9	C10	178.3(5)
C8	C9	C10	C11	178.0(5)
C9	C10	C11	C12	179.8(5)
C10	C11	C12	C13	-179.7(5)
C11	C12	C13	C14	179.9(5)
C12	C13	C14	C15	179.7(5)
C13	C14	C15	C16	-179.3(5)
C14	C15	C16	C17	179.0(5)

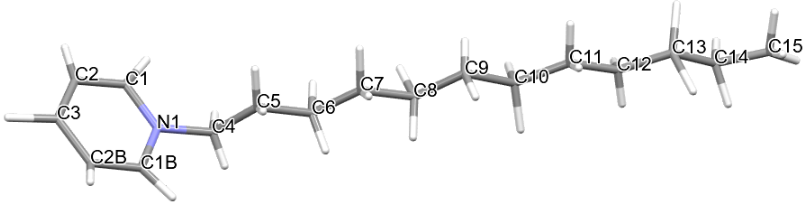

CSD refcode: POCHOE

Atom 1	Atom 2	Atom 3	Atom 4	Torsion/°
C1	N1	C4	C5	90
C1B	N1	C4	C5	-90
N1	C4	C5	C6	180
C4	C5	C6	C7	180
C5	C6	C7	C8	-180
C6	C7	C8	C9	-180
C7	C8	C9	C10	180
C8	C9	C10	C11	180
C9	C10	C11	C12	180
C10	C11	C12	C13	-180
C11	C12	C13	C14	180
C12	C13	C14	C15	180

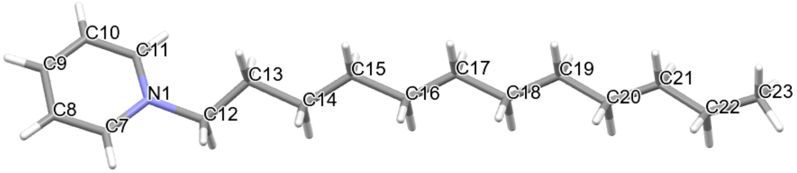

Atom 1	Atom 2	Atom 3	Atom 4	Torsion/°
C11	N1	C12	C13	34(1)
C7	N1	C12	C13	-153.0(8)
N1	C12	C13	C14	175.0(8)
C12	C13	C14	C15	176.1(9)
C13	C14	C15	C16	-179.3(9)
C14	C15	C16	C17	-177(1)
C15	C16	C17	C18	177(1)

CSD refcode: PULKUC

C16	C17	C18	C19	-178(1)
C17	C18	C19	C20	177(1)
C18	C19	C20	C21	-178(1)
C19	C20	C21	C22	178(1)
C20	C21	C22	C23	178(1)

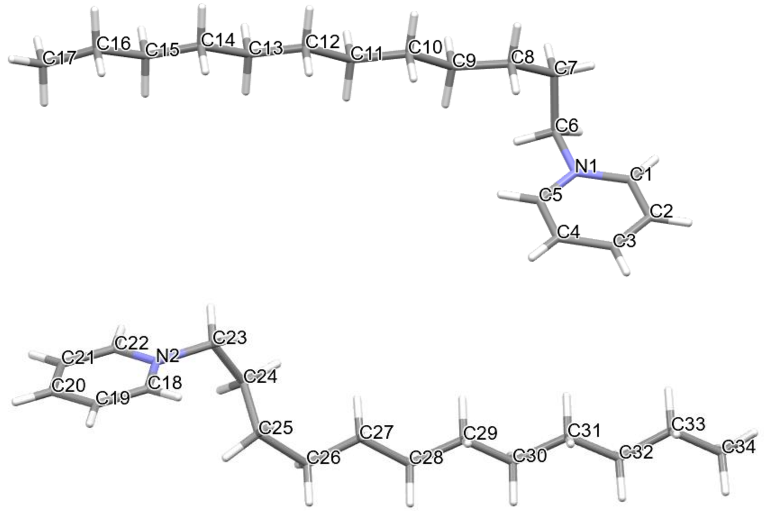

CSD refcode: WEQKEL

Atom 1	Atom 2	Atom 3	Atom 4	Torsion/°
C1	N1	C6	C7	-84.3(3)
C5	N1	C6	C7	94.9(3)
N1	C6	C7	C8	-69.8(3)
C6	C7	C8	C9	-66.3(3)
C7	C8	C9	C10	-176.8(2)
C8	C9	C10	C11	-177.7(2)
C9	C10	C11	C12	179.5(2)
C10	C11	C12	C13	179.9(2)
C12	C13	C14	C15	-177.0(2)
C13	C14	C15	C16	179.0(2)
C14	C15	C16	C17	-175.5(2)
C18	N2	C23	C24	94.2(3)
C22	N2	C23	C24	-87.6(3)
N2	C23	C24	C25	-68.5(4)
C23	C24	C25	C26	-160.8(3)
C24	C25	C26	C27	73.5(4)
C25	C26	C27	C28	178.3(4)
C26	C27	C28	C29	-171.7(4)
C27	C28	C29	C30	-178.4(4)
C28	C29	C30	C31	-176.7(4)
C29	C30	C31	C32	-178.6(4)
C30	C31	C32	C33	174.3(4)
C31	C32	C33	C34	-179.2(4)

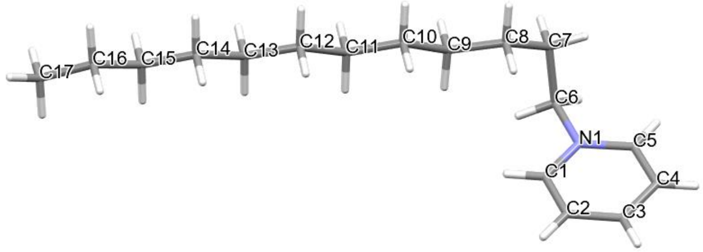

Atom 1	Atom 2	Atom 3	Atom 4	Torsion/°
C5	N1	C6	C7	-84.0(5)
C1	N1	C6	C7	95.1(4)
N1	C6	C7	C8	-69.8(5)
C6	C7	C8	C9	-65.6(5)
C7	C8	C9	C10	-176.7(4)
C8	C9	C10	C11	-178.3(4)
C9	C10	C11	C12	178.9(4)
C10	C11	C12	C13	179.3(4)

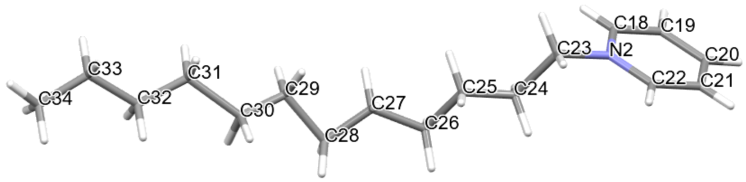

CSD refcode: WEQKIP

C11	C12	C13	C14	176.4(4)
C12	C13	C14	C15	-179.1(4)
C13	C14	C15	C16	179.1(4)
C14	C15	C16	C17	-176.6(4)
C18	N2	C23	C24	76.5(6)
C22	N2	C23	C24	-102.4(5)
N2	C23	C24	C25	-170.5(7)
C23	C24	C25	C26	175.1(7)
C24	C25	C26	C27	-172(1)
C25	C26	C27	C28	-168(1)
C26	C27	C28	C29	-163(1)
C27	C28	C29	C30	-153(2)
C28	C29	C30	C31	-175(1)
C29	C30	C31	C32	164(2)
C30	C31	C32	C33	170(1)
C31	C32	C33	C34	178(1)

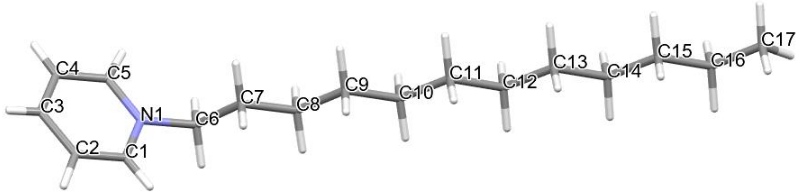

CSD refcode: ZAYVIE

Atom 1	Atom 2	Atom 3	Atom 4	Torsion/°
C1	N1	C6	C7	-78.5(5)
C5	N1	C6	C7	101.0(4)
N1	C6	C7	C8	179.0(3)
C6	C7	C8	C9	-180.0(4)
C7	C8	C9	C10	179.4(4)
C8	C9	C10	C11	179.9(4)
C9	C10	C11	C12	179.9(4)
C10	C11	C12	C13	179.9(4)
C11	C12	C13	C14	-179.9(4)
C12	C13	C14	C15	179.8(4)
C13	C14	C15	C16	-179.9(4)
C14	C15	C16	C17	179.3(5)

*Entry under refcode FIBJAD was discarded from the analysis due to a heavy positional disorder.

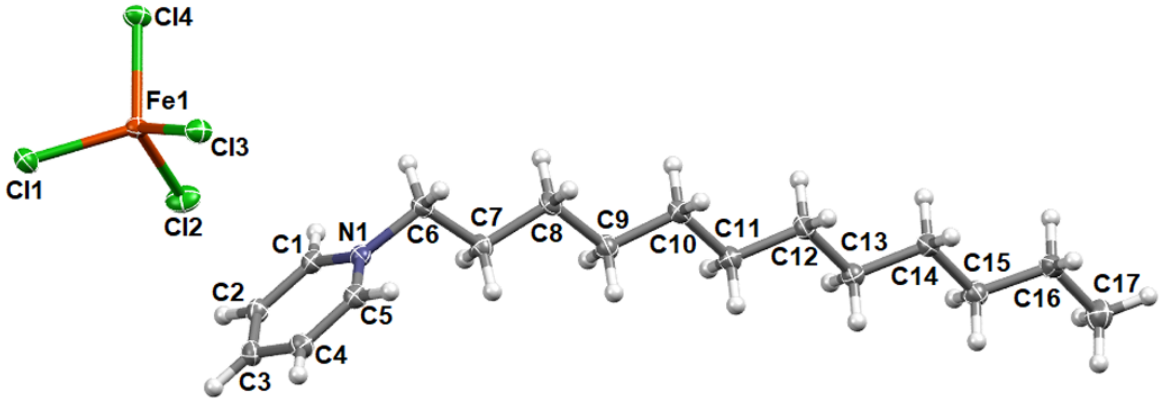

(a)

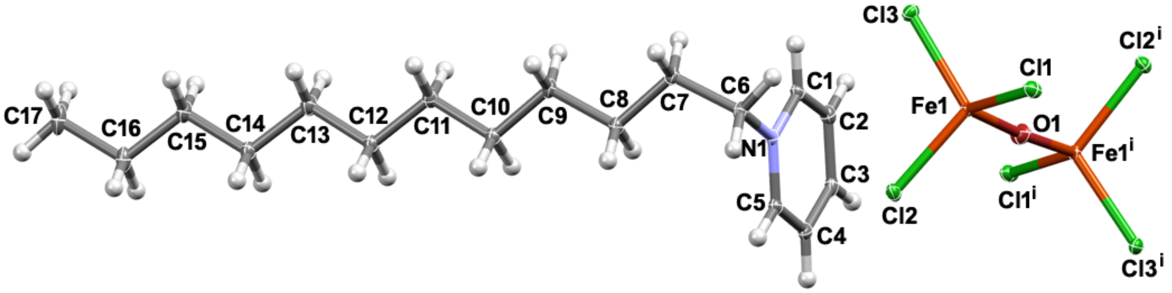

(b)

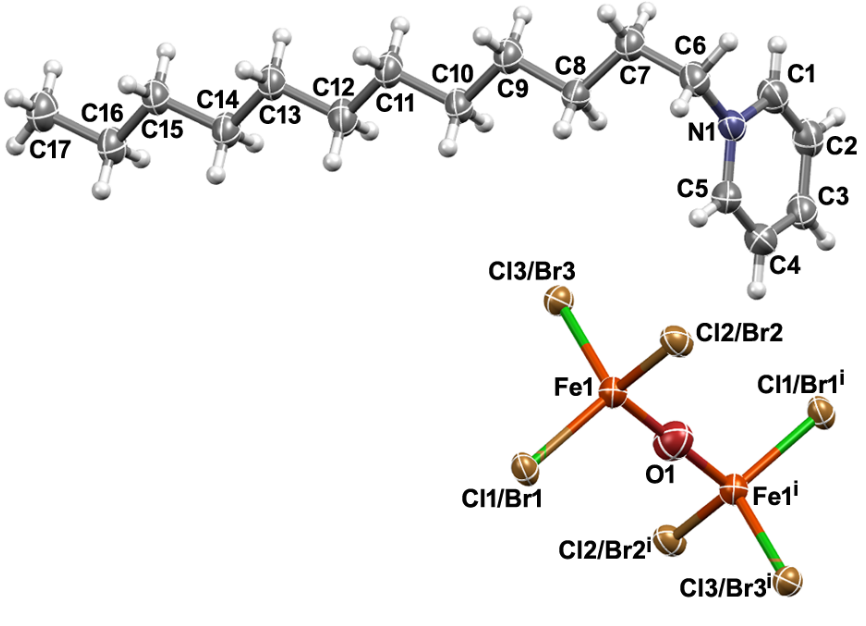

(c)

Figure S3. Molecular structures of: (a) $(C_{12}Py)[FeCl_4]$; (b) $(C_{12}Py)_2[Fe_2OCl_6]$; (c) $(C_{12}Py)_2[Fe_2OCl_3Br_3]$. In (c) the halogen positions are equally populated by Cl and Br (50:50 ratio). In: (b) *i* = -1/2-x,y,1-z; in (c) *i* = 2-x,2-y,1-z.

Table S3. Selected bond lengths, angles and hydrogen bond parameters in the crystal structure of $(C_{12}Py)[FeCl_4]$.

Atoms	Bond length/Å	Atoms	Bond angle/°	Atoms	Bond angle/°
Fe1–Cl4	2.1988(7)	Cl2–Fe1 –Cl4	108.64(3)	Cl3–Fe1 –Cl2	110.94(3)
Fe1–Cl2	2.1934(7)	Cl1–Fe1 –Cl4	109.47(3)	Cl3–Fe1 –Cl1	109.28(3)
Fe1–Cl1	2.1900(8)	Cl1–Fe1 –Cl	109.09(3)		
Fe1–Cl3	2.1884(8)	Cl3–Fe1 –Cl4	109.40(3)		
τ_4 value*	0.99				

* $\tau_4 = \frac{360° - (\alpha+\beta)}{141°}$; α, β are two largest angles in the four-coordinate species [31].

D–H···A	D-H	H···A	D···A	∠D–H···A	Symmetry code
C2–H2···Cl1	0.9500	2.8361	3.555(3)	133.3	-1/2-x,1.5-y,1-z

Table S4. Selected bond lengths, angles and hydrogen bond parameters in the crystal structure of $(C_{12}Py)_2[Fe_2Cl_6O]$.

Atoms	Bond length/Å	Atoms	Bond angle/°	Atoms	Bond angle/°
Fe1–Cl2	2.2226(5)	Cl2–Fe1–Cl3	109.679(18)	O1–Fe1–Cl3	110.94(6)
Fe1–Cl3	2.2234(5)	Cl2–Fe1–Cl1	108.397(18)	O1–Fe1–Cl1	109.137(16)
Fe1–Cl1	2.2282(5)	Cl3–Fe1–Cl1	108.525(18)	Fe1–O1–Fe1^i	175.83(12)
Fe1–O1	1.7629(3)	O1–Fe1–Cl2	110.11(6)	*i* = -1/2-x,+x,1-z	
τ_4 value*	0.99				

* $\tau_4 = \frac{360° - (\alpha+\beta)}{141°}$; α, β are two largest angles in the four-coordinate species [31].

D–H···A	D-H	H···A	D···A	∠D–H···A	Symmetry code
C1–H1···Cl1	0.9500	2.6000	3.4847(19)	155.00	1/2+x,1-y,z
C2–H2···Cl3	0.9500	2.7700	3.5491(19)	140.00	-x,1-y,1-z
C5–H5···Cl1	0.9500	2.6800	3.4747(19)	141.00	1/2+x,2-y,z

Table S5. Selected bond lengths, angles and hydrogen bond parameters in the crystal structure of $(C_{12}Py)_2[Fe_2Cl_3Br_3O]$.

Atoms	Bond length/Å	Atoms	Bond angle/°	Atoms	Bond angle/°
Br1/Cl1–Fe1	2.3408(19)	Br1/Cl1–Fe1–Br3/Cl3	109.83(7)	O1–Fe1–Br3/Cl3	111.08(8)
Br3/Cl3–Fe1	2.3493(19)	Br2/Cl2–Fe1–Br1/Cl1	109.55(8)	O1–Fe1–Br2/Cl2	107.79(7)
Br2/Cl2–Fe1	2.340(2)	Br2/Cl2–Fe1–Br3/Cl3	108.61(7)	Fe1–O1–Fe1^{i}	180
Fe1–O1	1.7534(14)	O1–Fe1–Br1/Cl1	109.94(8)	*i* = 2-x,2-y,1-z	
τ_4 value*	0.99				

* $\tau_4 = \frac{360° - (\alpha+\beta)}{141°}$ α, β are two largest angles in the four-coordinate species [31].

D–H···A	D-H	H···A	D···A	∠D–H···A	Symmetry code
C1–H1···Cl2/Br2	0.9500	2.7300	3.555(12)	145.00	1-x,1-y,1-z
C5–H5···Cl2/Br2	0.9500	2.8000	3.615(12)	144.00	2-x,2-y,1-z

Projection along Fe–Fe direction | Side view

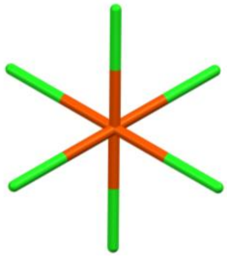

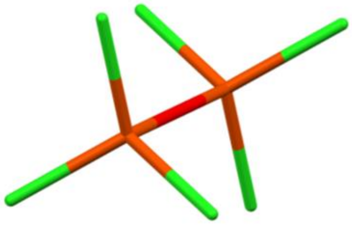

refcode: UBENUK
torsion angle Θ = 180 °

Staggered conformation

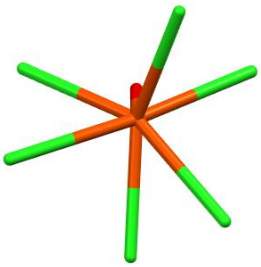

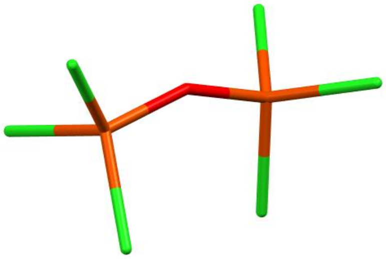

refcode: DEKHUW
torsion angle Θ = 45.6(3) °

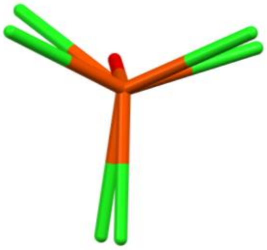

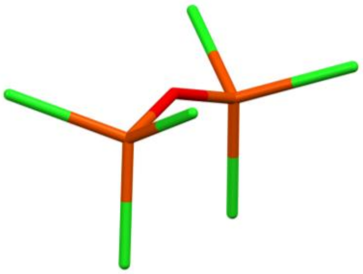

refcode: NADTAN
torsion angle Θ = 13.0(1) °

Skew conformation

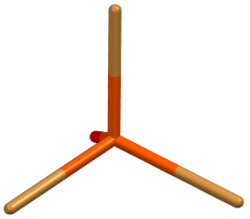

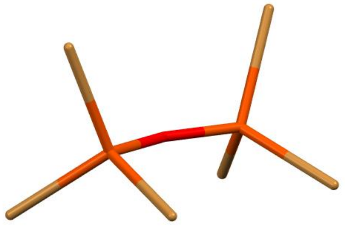

refcode: KOVMOX
torsion angle Θ = 0 °

Eclipsed conformation

Figure S4. Illustrative examples of conformations observed in of conformations observed in a $[Fe_2X_6O]^{2-}$ containing structures (X = Cl, Br).

Solution properties

Values of the critical aggregation concentrations (denoted as cac_1 and cac_2) were determined from the intersection of the two straight lines drawn above and below the observed breaks in the electrical conductivity (κ) *vs.* surfactant concentration (c), using a linear regression analysis.

The apparent degree of counterion dissociation from the micelle/solution interface (α) was calculated as the ratio of the slopes above and below the cac_2 in κ *vs.* c curves [70]. The apparent degree of counterion binding to the micelle/solution interface (β) was calculated from the relation:

$$\beta = 1 - \alpha \quad \text{(eq. S1)}$$

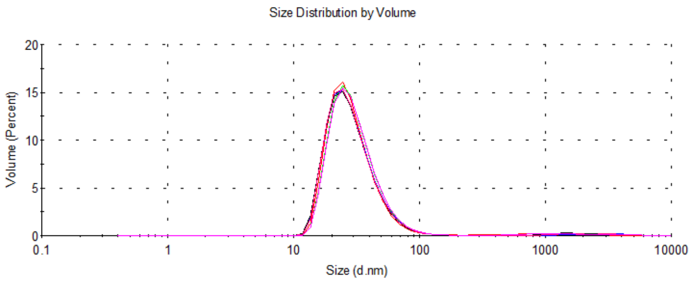

(a) 1 mM

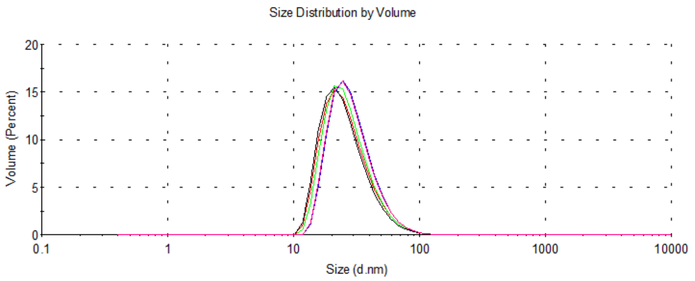

(b) 5 mM

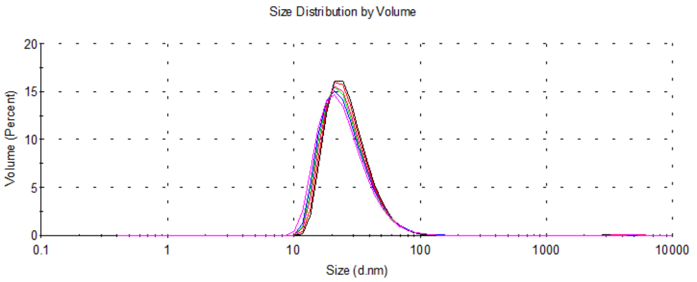

(c) 10 mM

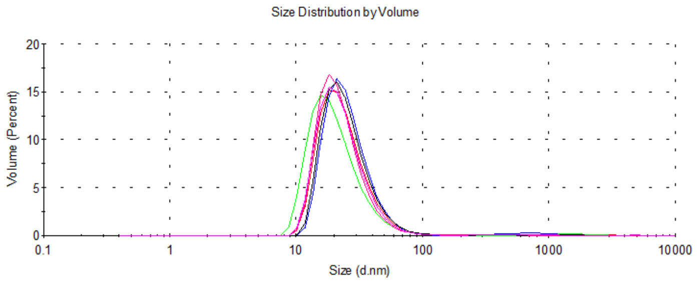

(d) 35 mM

Figure S5. Measured volume size distributions in (C_{12}Py)[$FeCl_4$] systems at different concentrations.
